# Digital Shared Decision-Making Interventions in Mental Healthcare: A Systematic Review and Meta-Analysis

**DOI:** 10.3389/fpsyt.2021.691251

**Published:** 2021-09-06

**Authors:** Tobias Vitger, Lisa Korsbek, Stephen F. Austin, Lone Petersen, Merete Nordentoft, Carsten Hjorthøj

**Affiliations:** ^1^Competence Center for Rehabilitation and Recovery, Mental Health Center Ballerup, Mental Health Services - Capital Region of Denmark, Copenhagen, Denmark; ^2^The Mental Health Centre Odense, Mental Health Services in the Region of Southern Denmark, Esbjerg, Denmark; ^3^Psychiatric Research Unit, Psychiatry Region Zealand, Slagelse, Denmark; ^4^Copenhagen Research Center for Mental Health - CORE, Mental Health Center Copenhagen, Copenhagen University Hospital, Copenhagen, Denmark; ^5^Department of Public Health, Section of Epidemiology, University of Copenhagen, Copenhagen, Denmark

**Keywords:** shared decision-making, systematic review and meta-analysis, mental health, digital health (eHealth), patient activation

## Abstract

**Background:** Shared decision-making (SDM) in mental healthcare has received increased attention as a process to reinforce person-centered care. With the rapid development of digital health technology, researchers investigate how digital interventions may be utilized to support SDM. Despite the promise of digital interventions to support SDM, the effect of these in mental healthcare has not been evaluated before. Thus, this paper aims to assess the effect of SDM interventions complimented by digital technology in mental healthcare.

**Objective:** The objective of this review was to systematically examine the effectiveness of digital SDM interventions on patient outcomes as investigated in randomized trials.

**Methods:** We performed a systematic review and meta-analysis of randomized controlled trials on digital SDM interventions for people with a mental health condition. We searched for relevant studies in MEDLINE, PsycINFO, EMBASE, CINAHL, and the Cochrane Central Register of Controlled Trials. The search strategy included terms relating to SDM, digital systems, mental health conditions, and study type. The primary outcome was patient activation or indices of the same (e.g., empowerment and self-efficacy), adherence to treatment, hospital admissions, severity of symptoms, and level of functioning. Secondary outcomes were satisfaction, decisional conflict, working alliance, usage, and adherence of medicine; and adverse events were defined as harms or side effects.

**Results:** Sixteen studies met the inclusion criteria with outcome data from 2,400 participants. Digital SDM interventions had a moderate positive effect as compared with a control condition on patient activation [standardized mean difference (SMD) = 0.56, CI: 0.10, 1.01, *p* = 0.02], a small effect on general symptoms (SMD = −0.17, CI: −0.31, −0.03, *p* = 0.02), and working alliance (SMD = 0.21, CI: 0.02, 0.41, *p* = 0.03) and for improving decisional conflict (SMD = −0.37, CI: −0.70, −0.05, *p* = 0.02). No effect was found on self-efficacy, other types of mental health symptoms, adverse events, or patient satisfaction. A total of 39 outcomes were narratively synthesized with results either favoring the intervention group or showing no significant differences between groups. Studies were generally assessed to have unclear or high risk of bias, and outcomes had a Grading of Recommendations Assessment, Development and Evaluation (GRADE) rating of low- or very low-quality evidence.

**Conclusions:** Digital interventions to support SDM may be a promising tool in mental healthcare; but with the limited quality of research, we have little confidence in the estimates of effect. More quality research is needed to further assess the effectiveness of digital means to support SDM but also to determine which digital intervention features are most effective to support SDM.

**Systematic Review Registration:** PROSPERO, identifier CRD42020148132.

## Introduction

Digital health technology has become an integrated part of the global healthcare system and is continuously developing and growing. Within mental healthcare, traditional means of care are being complemented by health technology such as smartphone decision aids, web-based self-management systems, or online support groups. As technology develops, new possibilities arise; and the World Health Organization advocated through the global strategy on digital health for 2020–2024 to use digital technology for more person-centered healthcare (1). Person-centered care focuses on placing people at the center of their healthcare, and technology may complement this in various ways such as supporting people to become more aware of their health and needs.

Researchers within mental healthcare have increasingly turned their attention to shared decision-making (SDM) as a process to reinforce person-centered care (2). SDM can be defined as a process involving at least two people (e.g., patient and provider) who share information, discuss options, and collaborate to reach a mutual decision (3). SDM aims to ensure that both patient and provider are actively involved in decision-making processes and that their unique competences are utilized. Providers have an expertise in information on symptoms management, treatment options, and potential benefits or side effects; while patients are experts on their needs, preferences, goals, and values. SDM may be affected by mechanics surrounding the patient and provider such as their individual engagement, working alliance, and mutual understanding of one another but also the risk associated with the decision. If done successfully, SDM may increase autonomy, self-management, working alliance, satisfaction, and quality of care (4). For SDM to be successful, both the patient and provider must be engaged in the patients' care. Patients have indicated that being an active partner and embracing the same qualities as one would expect from their provider is necessary for the success of SDM (e.g., honesty, responsibility, and trust) (3). Patients in mental healthcare have also indicated that they want to be active participants when making health decisions (5). Still, SDM has not been widely implemented in clinical practice with barriers such as time constraints at consultations, providers believing they can guess how the patients wish to be involved, or uncertainty of how to fit SDM into the workflow (2, 6). In addition, recent research notes that SDM may be easier to incorporate when making a decision has a low personal risk and may be more difficult to incorporate when decisions have a higher risk such as adjusting one's medication (7). A systematic review—covering 33 studies on including patients in decision-making—reported that a minority of healthcare providers consistently attempted to facilitate patient involvement, and even fewer adjust care to patient preferences (6). The review highlights SDM interventions as a means to promote patient-involving behaviors but that the responsibility of facilitating SDM cannot lie solely with the provider—decision aids and communication tools may serve as part of the solution (6). Therefore, to facilitate the process of SDM, research has begun to investigate how digital interventions may be utilized to support SDM and potentially address some of its barriers. Using technology as the tool and SDM as the process, randomized controlled trials (RCTs) are investigating whether digital SDM interventions are effective at promoting person-centered care.

Previous systematic reviews and meta-analyses have found that SDM interventions have a small effect on empowerment for people with psychosis and that SDM may increase provider facilitation of patient involvement (8, 9), while meta-analyses on the effect of digital interventions for mental health have found an effect at improving symptoms (10). However, a systematic assessment of the effect of digital interventions to support SDM in mental healthcare has not been conducted before. Thus, this paper aims to assess the effect of SDM interventions complimented by digital technology in mental healthcare for promoting person-centered care. Using subgroup analyses, we explored whether the effect was dependent on the type of digital intervention, age, or mental health condition. The results of these meta-analyses may guide future research and stakeholders in how digital technology may complement SDM in mental healthcare.

## Methods

This systematic review and meta-analysis followed the PRISMA statement and adhered to the registered online protocol at PROSPERO (CRD42020148132) (11).

### Definitions

We defined SDM in this review as a process with three main components: (1) sharing information; (2) discussing treatment options; and (3) reaching a mutual decision that both parties can agree upon. Around these three components lie several surrounding mechanisms affecting this process such as learning about the patient, supporting the patient to initiate discussions with the provider, or evaluating the decision. The process of SDM and its surrounding mechanisms is illustrated in Supplementary Material 1. This definition is based on previous research investigating patients' understanding of SDM and also a systematic review on the most common components of SDM models (3, 12). In this review, an SDM intervention is an intervention that supports at least one of the three main components of the SDM process.

Digital interventions were defined as information and/or communication technology delivered via phones, computers, personal digital assistants, or other similar devices. Interventions did not have to be internet-based.

Mental health conditions were defined in concordance to the *Diagnostic and Statistical Manual of Mental Disorders* (DSM) and *International Classification of Diseases* (ICD).

### Search Strategy

We conducted a systematic literature search of the following databases up to March 2021: MEDLINE, CINAHL, EMBASE, PsycINFO, and the Cochrane Central Register of Controlled Trials. The PICO framework was used to develop the search strategy. Our search terms focused on SDM, digital health technology, mental health, and RCTs. The complete search strategy is listed in the Supplementary Materials. Our search terms on SDM were developed based on existing Cochrane reviews on SDM (9, 13). Due to the complexity of SDM, our search terms focused on person-centered terms (e.g., patient involvement), technique style (e.g., decision aids), and relationship components (e.g., working alliance). Search terms on digital health technology focused on components such as e-Health, m-Health, and information technology. Search terms on mental health were broad and attempted to reach all mental health conditions. The reference lists of retrieved studies were checked to identify further eligible studies.

### Study Selection Criteria

Only RCTs presenting original data were included in the review. For a study to be included in the review, 50% of the participants needed to have a mental health condition as defined by the DSM and ICD. Besides having a mental health condition, there were no restrictions regarding clinical or demographic characteristics of the participants. Exclusion criteria were studies focusing on relatives rather than the patient or provider. For an intervention to be included, it had to cover one of the three main components of SDM and use a digital tool for people with a mental health condition (as defined above).

The search strategy was developed by the author TV and approved by CH and LK (see Supplementary Material 2 for the search string). All identified studies were extracted and exported into Zotero reference manager software by TV. All identified studies were title screened by TV against inclusion/exclusion criteria to determine eligibility for selection. The abstract screen and full-text assessments were independently performed by CH and TV with a 74% agreement. In case of disagreements, a third reviewer (LK) was included in the discussion.

### Data Extraction

For included studies, the following data were extracted by TV into predefined tables: year of publication, sample size, mental health condition, type of intervention, duration of intervention, type of outcome, results (number of events, means, and SD), control condition, type of setting, and baseline demographics (age, gender, and the highest educational level). The authors of the retrieved papers were contacted if clarification was needed or if data were not accessible from the article.

### Statistical Analyses

All analyses were conducted by Review Manager 5.3.5, using random-effects model to account for heterogeneity. The total difference in changes on measurements for patient activation or indices of the same between digital interventions and controls were pooled to compute the overall effect size of the digital interventions with 95% confidence intervals. TV and CH assessed the risk of bias in the included studies using Cochrane Collaboration's Risk of Bias tool. This tool assesses studies six areas, ranking each area as high, low, or unknown for risk of bias. The areas are sequence generation, allocation sequence concealment, blinding of participants and personnel, blinding of outcome assessment, incomplete outcome data, and selective outcome reporting. In addition, TV and CH used the Grading of Recommendations Assessment, Development and Evaluation (GRADE) system to assess the quality of evidence of each outcome by downgrading from high by one level for each serious issue identified in the domains: risk of bias, inconsistency, indirectness, imprecision, and publication bias (14). In case of a disagreement on the assessment of the studies' risk of bias and the outcomes' GRADE score, a third reviewer was included in the discussion. As stated in our protocol, subgroup analysis was performed for type of intervention (web-based, PC software, or smartphone/tablet application) and diagnosis (11). However, due to discrepancies in the length of the interventions, subgroup analysis on the duration of intervention was also included and was divided into short-term (<3 months) and long-term interventions (>3 months). The cutoff at 3 months was chosen to ensure that trials would be divided somewhat equally and that subgroup analysis on duration would be feasible. For outcomes only occurring in one trial or outcome data not appropriate for a meta-analysis (and where the corresponding author was unable to assist), we did a narrative synthesis. The synthesis was done by counting the numbers of trials reporting a significant positive effect, no effect, and a negative effect for each outcome.

### Changes From Protocol

Decisional conflict was not originally planned as an outcome for our meta-analysis according to our protocol; however, after discovering several of the studies measuring this area and considering its relation to SDM, it was included (15–18).

## Results

**Figure 1:**
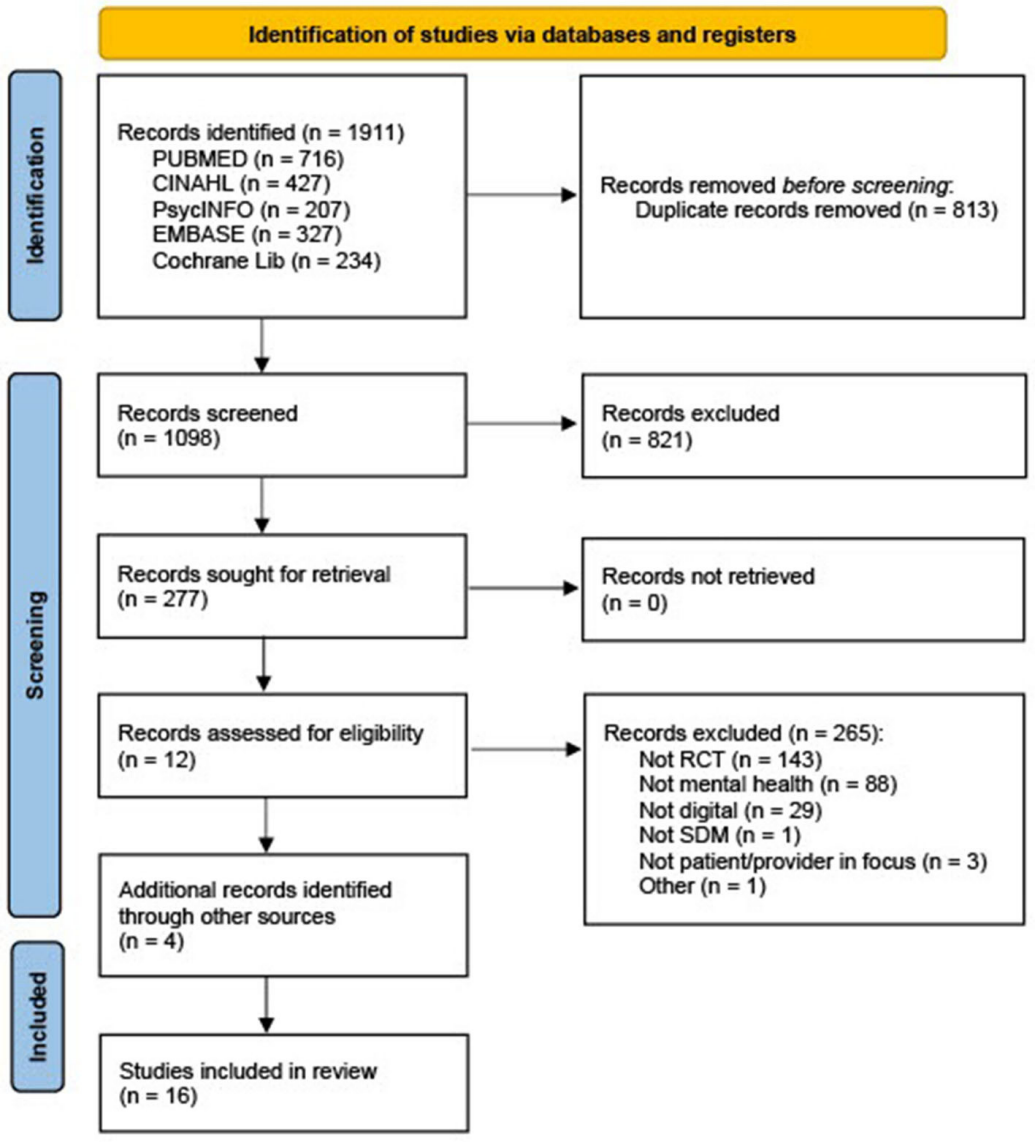
PRISMA flow diagram (19).

The search resulted in 1,911 references, 1,098 after duplicates were removed and 277 after title screening. After abstract screen and full-text assessment, 12 articles were assessed as eligible. Four additional articles were included after reading protocols from the initial search where authors of unpublished results were contacted. Thus, 16 RCTs investigating digital SDM interventions within mental health were included in the meta-analysis. The progress of including and excluding articles is shown in the PRISMA flow diagram Figure 1.

### Study Characteristics

Characteristics of the 16 study populations, aim, duration, comparison treatment, and outcome measures are presented in Table 1.

**Table 1:** Characteristics of the 16 trials.

**References, Location**	***N***	**Mental health condition**	**Duration**	**Aim**	**Comparison treatment**	**Outcome measure**
Steinwach et al. (20), USA	50	Schizophrenia	1 day	A web-based intervention to support patients navigate areas of care supplemented by video clips of actors simulating a patient discussing treatment concerns. Goal was to increase the likelihood that patients will initiate discussion with their therapist	An educational video about schizophrenia treatment	Visit duration; Patient contribution; Reduce amount of clinician talk; Amount of questions asked
Van der Krieke et al. (21), The Netherlands	73	Psychosis	6 weeks	A web-based information and a decision tool aimed to support patients in acquiring an overview of their needs and appropriate treatment options	Treatment as usual	Patient perceived involvement (two subscales): Satisfaction with communication (COMRADE); Confidence in decision (COMRADE); Client Satisfaction Questionnaire (CSQ-8); Satisfaction With the Web-Based Decision Aid
Perestelo et al. (17), Spain	147	Major depressive disorder	1 day	A web-based decision aid aimed to improve users knowledge and promote their active participation in health-care decisions	Treatment as usual	Knowledge about treatment options (authors scale); Decisional conflict (DCS), Treatment intention; Preference for participation in decision making (Control Preference Scale)
Metz et al. (16), The Netherlands	200	Personality, Anxiety or Mood disorder	2 months	A website aimed to support patients in preparing themselves and be more able to actively participate in the dialogue with their clinicians about choices in treatment	Treatment as usual	Decisional Conflict (DCS); Patient participation (PPQ); SDM process (SDM-Q-9); Working alliance (PRDRQ-9); Symptom severity (SQ-48); No-show and Drop out
Moncrieff et al. (22), UK	60	Psychosis, schizophrenia, schizoaffective disorder, delusional disorder or a mood disorder with psychotic symptoms and currently taking antipsychotic medication	3 months	A web-based medication review tool to gain information about psychotic conditions, medication and support people to consider when to discuss and make decisions about medication with professionals	Treatment as usual	Decision Self-Efficacy Scale (DSES); Client Satisfaction Questionnaire (CSQ-8); Drug Attitude Inventory 10 (DAI-10); Liverpool University Neuroleptic Side Effect Rating Scale (LUNSERS); Brief Positive and Negative Syndrome Scale (Brief PANSS); Medication Adherence Questionnaire
Priebe et al. (23), Spain, The Netherlands, Sweden, UK, Germany, Switzerland	507	Schizophrenia or related disorders	12 months	A computer-mediated procedure, DIALOG, to ensure that a range of life domains and treatment aspects were consistently addressed and patients' perspectives always elicited	Treatment as usual	Quality of life (MANSA); Unmet needs (CANSAS); Satisfaction (CSQ-8); Symptoms (PANSS)
Woltmann et al. (24), USA	80	Schizophrenia, schizoaffective, bipolar disoder, major depressive disorder, posttraumatic stress disorder	4 days	An electronic decision support system to support client involvement in goal setting and to assist clients and case managers in engaging in shared decision-making	Treatment as usual	Satisfaction with the care planning process; Knowledge of the care plan; Case manager satisfaction with the care planning process
Manthey (25), USA	110	Schizophrenia, bipolar, or major depression	3 months	An electronic decision support aid to conduct self-assessments of their strengths, identify personal recovery goals, link their strengths to their goals, and identify initial tasks toward goal completion	Treatment as usual	Empowerment (Empowerment scale); Self-Determination Scale (subscales: Awareness of self and perceived choice); Stage of Recovery (SIS-R)
Campbell et al. (26), USA	84	Schizophrenia, bipolar, or major depression	5 months	A computer program, CommonGround, that included videos of consumers who talk about their recovery, answers questions concerning medication usage and decisional uncertainty etc. A report is generated that the patient can bring to his/her consultation to help set the agenda by focusing on the consumer's values and decisional uncertainty	Treatment as usual	Measure of Patient-Centered Communication (MPPC); The Patient Perception of Patient-Centeredness Questionnaire (PPPC) for patient and provider
Edbrooke-Childs et al. (27), UK	62	Unclear – Children and young people from 8 Child mental health services	3 months	A smartphone app, Power up, with the aim to promote patient activation. Used to record questions, plans, decisions, and diary entries and supports young people to identify individuals in their support network	Treatment as usual	The Patient Activation Measure (PAM-MH); (2) CollaboRATE; Shared decision-making Questionnaire (SDM-Q-9); Youth Empowerment Scale—Mental Health; The Strengths and Difficulties Questionnaire; The Experience of Service Questionnaire
Vigod et al. (18), Canada	96	Major depressive episode, Generalized anxiety disorder, Panic disorder, Social anxiety disorder, Obsesssive compulsive disorder, Posttraumatic stress disorder	1 month	A web-based tool aimed to increase knowledge, provide evidence based information on medication, help patient consider how relationships with family, partners, providers etc. may impact their decisions	Online information sheet comprising publicly available information	Decisional Conflict Scale (DCS); Symptoms (depression and anxiety); Knowledge
MacInnes et al. (28), UK	112	Schizophrenia and Schizoaffective disorders and other mental health disorders	6 month	A tablet app to assess and record their satisfaction with life and treatment domains–patient and nurse would together go over relevant domains	Providers were encouraged to meet control patients with the same frequency as intervention and discuss difficulties but without the structured communication approach as in the intervention group	Quality of Life (MANSA); Engagement with Services (HAS); Ward Climate; Patient Satisfaction [Forensic Satisfaction Scale (FSS)]; Recovery [Process of Recovery Questionnaire (QPR)]; Nurse Stress; Disturbed behavior; Satisfaction with intervention; service user perspectives, and experiences of the study
Kravitz (29), USA	391	Depression	3 months	A tailored interactive multimedia computer program providing patients with feedback tailored to symptoms, visit agenda, depression causal attributions, treatment preferences, self-efficacy for communicating with healthcare providers, and depression stigma	A sleep hygiene video	Receiving an antidepressant recommendation or a mental health referral; Patient-physician communication self-efficacy; Whether the patient reported asking the provider for information about depression; Scores on the PHQ-8; SF-12 Version 2.0 Mental Health Component (MCS-12); Physical Health Component Summary Scores (PCS-12)
Priebe (30), UK	179	Psychosis	6 months	A tablet app aimed to provide a way to deal with concerns raised by the patient and equip the clinician and patient with a method to explore and deal with problems	The same app as the intervention, however, it was used at the end of the consultation and used independently rather than collaboratively and without further discussion	Quality of life (MANSA); Unmet needs (CANSAS); Satisfaction (CSQ-8); Self-efficacy (GSE); Mental well-being [Warwick-Edinburgh Mental Well-Being Scale (WEMWBS)]; Symptoms (PANSS); Therapeutic relationship [Scale for Assessing Therapeutic Relationships in Community Mental Health Care (STAR-P And STAR-C)]; Social functioning
Fisher et al. (15), Australia	196	Bipolar II disorder	3 months	A web-based decision-aid to improve treatment decision-making regarding options for relapse prevention in Bipolar disorders	Access to publicly available, evidence-based information on treatment options for bipolar disorder	Symptoms (Bipolar and/or anxiety symptoms); Decisional conflict; Knowledge of treatment options; Feeling prepared for decision making; Decisional regret
Yamaguchi et al. (31), Japan	53	Schizophrenia (70%) and other psychiatric diagnoses	6 months	A computer program with peer support to support shared decision-making. A report is generated that the patient can bring to his/her consultation to help set the agenda by focusing on the consumer's values and decisional uncertainty	Treatment as usual	Shared decision-making (SDM-18); cale To Assess Therapeutic Relationships in Community Mental Health Care (STAR-C and STAR-P); Level of communication with doctor (IPC); Patient activation (PAM); (5) Satisfaction (CSQ); Symptoms (BPRS–Brief Psychiatric Rating Scale); Level of functioning (GAF); Medication side effects (Drug Induced Extra-Pyramidal Symptoms Scale); Adherence to medication (MMAS–Morisky Medication Adherence Scale); Quality of Life–WHO-QOL; Recovery (SISR–Self-Identified Stage of Recovery)

Overall, 2,400 participated in the 16 trials with a mean sample size of 150 ranging from 50 to 507 participants. Mean age for the participants was 40 years (SD = 8.5) ranging from 15 to 51 years. The proportion of females was 49% across all studies ranging from 14 to 100%. The mean duration of the interventions was approximately 3.4 months ranging from 1 day to 12 months. Because of this variation, studies were divided into either short-term intervention (3 months or less) or long-term intervention (more than 3 months): 11 trials were categorized as short-term interventions (15–18, 20–22, 24, 25, 27, 29) and five trials as long-term interventions (23, 26, 28, 30, 31). Seven studies investigated a system accessible through a website (15–18, 20–22), six studies investigated a computer software program (23–26, 29, 31), one study investigated a smartphone application (27), and two studies investigated a tablet application (28, 30). Half of the 16 studies reported using some form of clinician training and that the intervention was used in conjunction with the provider or a peer worker (16, 22–24, 26, 28, 30, 31). Studies that did not actively involve the clinicians mentioned, however, that patients were encouraged to talk with their provider about the intervention. Eleven studies mentioned an inclusion criterion of mental health condition, four studies mentioned recruiting from mental health services (15, 18, 24, 26), and one study mentioned recruiting from primary care; and a majority of their participants were assessed as depressed by the research team (29). Ten studies recruited exclusively or primarily participants with psychosis, schizophrenia, or related disorder (20–26, 28, 30, 31). Three studies recruited primarily participants with depression or depressive symptoms (17, 18, 29), one study recruited participants with a personality disorder (16), one study focused entirely on bipolar disorder (15), and one study only mentioned that participants were recruited from a mental health setting without specifying the type of mental health condition (27).

### Risk of Bias

**Figure 2:**
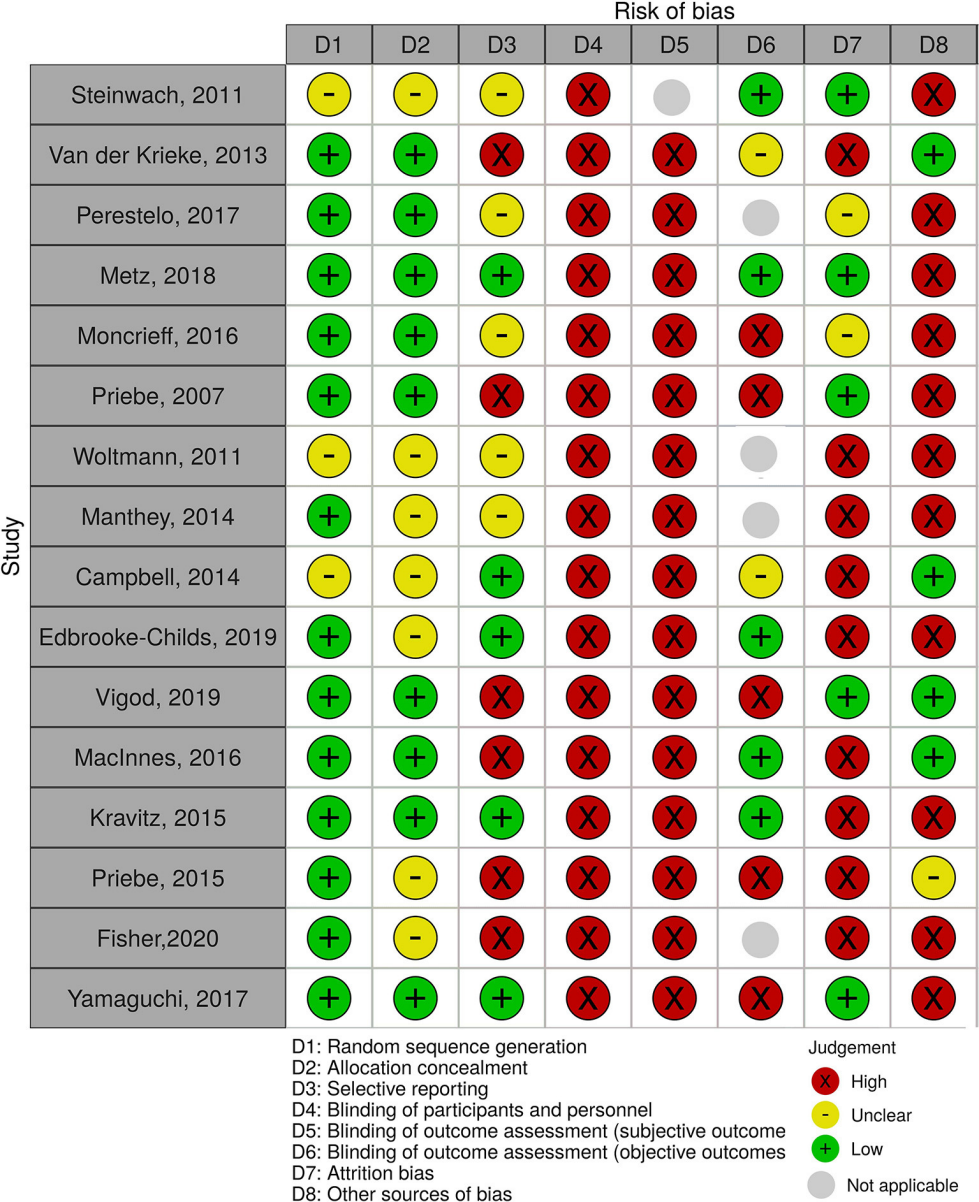
Risk of bias of the 16 trials.

All included studies were assessed for risk of bias using the Cochrane Collaboration's Risk of Bias tool on the following domains: random sequence generation, allocation concealment, selective reporting, blinding of participants and personnel, blinding of outcome assessments (objective and subjective), attrition bias, and other bias. The risk of bias for each study is presented in Figure 2 showing that the most frequent risk factor for bias was blinding of participants and blinding of subjective outcome assessment. Furthermore, more than half of the studies were at high risk of bias in terms of attrition bias and other bias.

### Meta-Analysis on the Effects of the Primary Outcomes

#### Patient Activation or Indices of the Same

**Figure 3:**
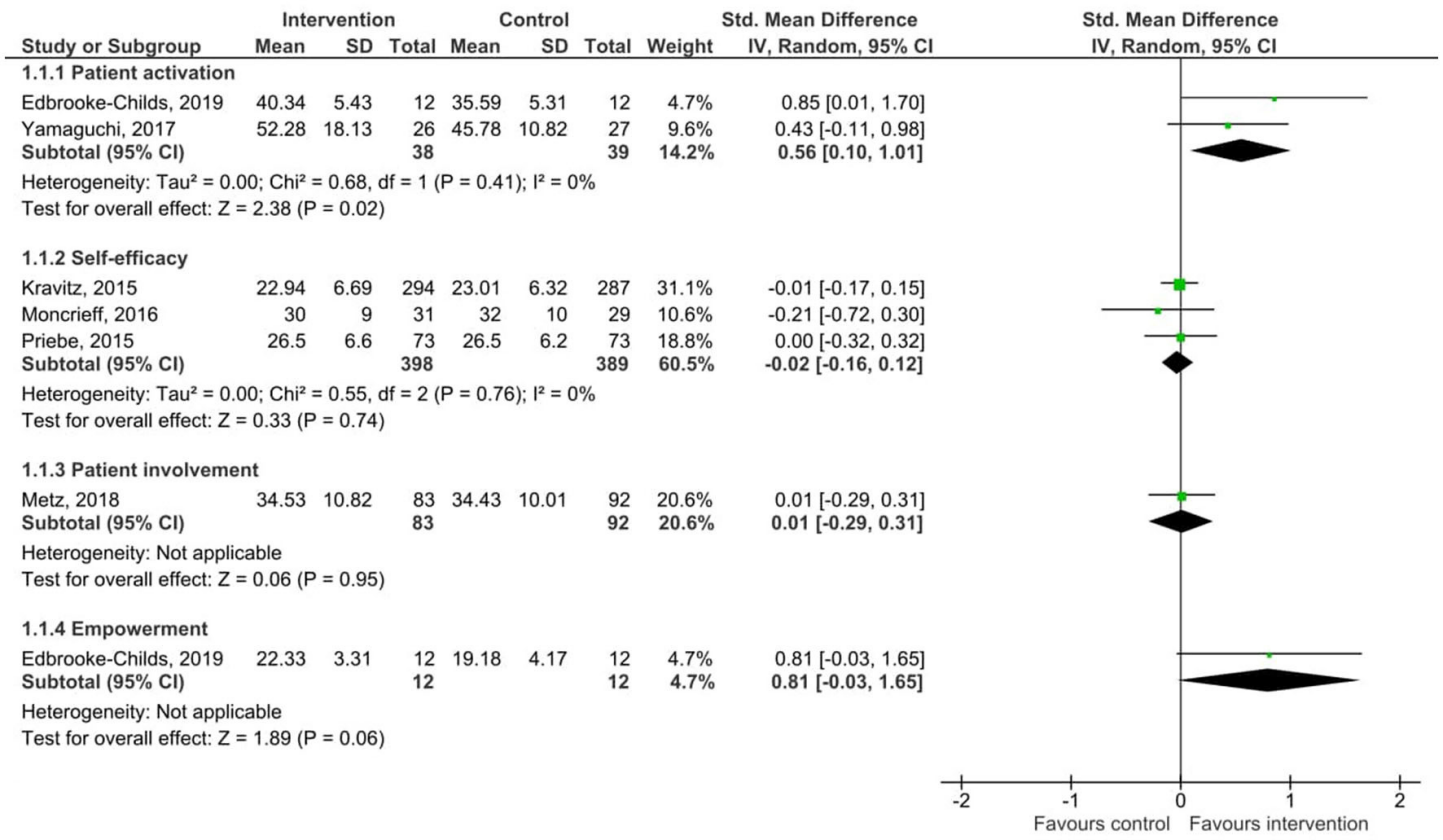
Forest plot for patient activation and indices of the same.

Results of the pooled effect size from digital SDM interventions on patient activation, self-efficacy, empowerment, and patient involvement with the individual effect of each intervention are presented in Figure 3. The random-effects meta-analysis revealed a moderate significant effect of digital SDM interventions to promote patient activation in comparison with a control group (two studies, *N* = 77, standardized mean difference (SMD) = 0.56, CI: 0.10, 1.01, *p* = 0.02) (27, 31). Variation across trials due to heterogeneity was not present (Chi^2^ = 0.68, *p* = 0.41, *I*^2^ = 0%). There was no significant effect on self-efficacy (three studies, *N* = 787, SMD = −0.02 CI: −0.16, 0.12, *p* = 0.74) (22, 29, 30). Variation across trials due to heterogeneity was not present (Chi^2^ = 0.55, *p* = 0.76, *I*^2^ = 0%). Meta-analysis on empowerment and patient involvement was not applicable due to only one study respectively reported data on these areas. Still, the results of these single studies are included in Figure 3, indicating no significant effect of digital SDM interventions on empowerment (SMD = 0.81, CI: −0.03, 1.65, *p* = 0.06) (27) or patient involvement (SMD = 0.01, CI: −0.29, 0.31, *p* = 0.95) (16). Due to differences between these aspects and the fact that one study measured both activation and empowerment, the total score in Figure 3 was removed to avoid study participants being counted twice.

#### Symptoms

**Figure 4:**
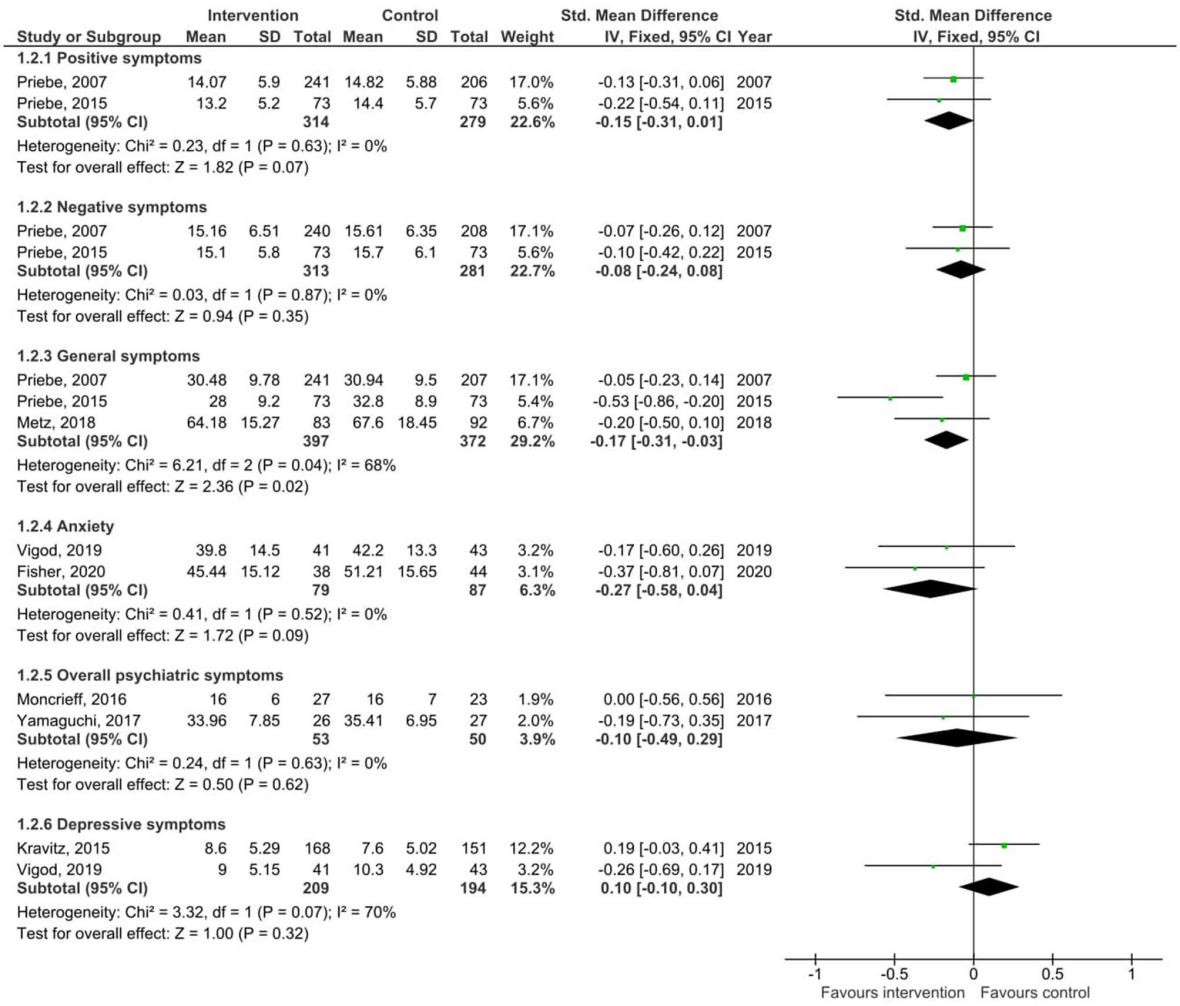
Forest plots for symptoms.

Results of the pooled effect size from digital SDM interventions on symptoms together with the individual effect of each intervention are presented in Figure 4. The random-effects meta-analysis revealed a small significant effect of digital SDM interventions to improve general symptoms (three studies, *N* = 769, −0.17, CI: −0.31, −0.03, *p* = 0.02) (16, 23, 30) but revealed no effect on positive (two studies (same research group), N = 593, SMD = −0.15, CI: −0.31, 0.01, *p* = 0.07) (23, 30), negative symptoms [two studies (same research group), *N* = 594, SMD = −0.08, CI: −0.24, 0.08, *p* = 0.35] (23, 30), overall psychiatric symptoms (two studies, *N* = 103, SMD = −0.10, CI: −0.49, 0.29, *p* = 0.62) (22, 31), depressive symptoms (two studies, *N* = 403, SMD = 0.10, CI: −0.10, 0.30, *p* = 0.32) (18, 29), or anxiety (two studies, *N* = 166, SMD = −0.27, CI: −0.58, 0.04, *p* = 0.09) (15, 18). Due to differences between the types of symptoms and that studies appeared more than once, the total score in Figure 4 was removed to avoid study participants being counted twice.

Variation across trials due to heterogeneity was not present for positive symptoms (Chi^2^ = 0.23, *p* = 0.63, *I*^2^ = 0%), negative symptoms (Chi^2^ = 0.03, *p* = 0.87, *I*^2^ = 0%), overall psychiatric symptoms (Chi^2^ = 0.24, *p* = 0.63, *I*^2^ = 0%), or anxiety (Chi^2^ = 0.41, *p* = 0.52, *I*^2^ = 0%) but was present for general symptoms (Chi^2^ = 6.21, *p* = 0.04, *I*^2^ = 68%) and depressive symptoms (Chi^2^ = 3.32, *p* = 0.07, *I*^2^ = 70%). Egger's test was not performed due to the small sample of studies.

#### Adverse Events

**Figure 5:**
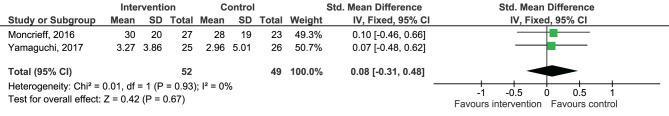
Forest plot for adverse events defined as harms or side effects.

Results of the pooled effect size from digital SDM interventions on adverse events defined as harms or side effects together with the individual effect of each intervention are presented in Figure 5. Only measurements of side effects of medication were identified from the studies. The random-effects meta-analysis revealed no significant effect of digital SDM interventions to improve side effects induced by medication (two studies, *N* = 102, SMD = 0.08, CI: −0.31, 0.48, *p* = 0.67) (22, 31). Variation across trials due to heterogeneity was not present (Chi^2^ = 0.01, *p* = 0.93, *I*^2^ = 0%). Subgroup analysis for the primary outcomes showed no significant differences, which are included in the Supplementary Materials 3–8).

### Meta-Analysis on the Effects of the Secondary Outcomes

**Figure 6:**
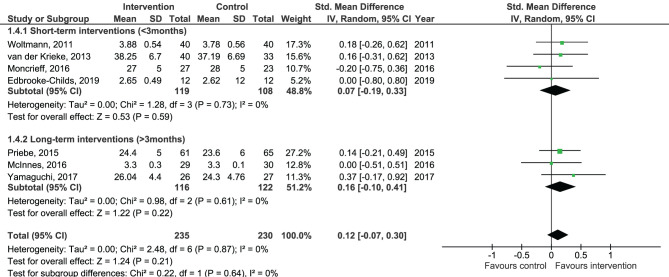
Forest plot for patient satisfaction by duration.

**Figure 7:**
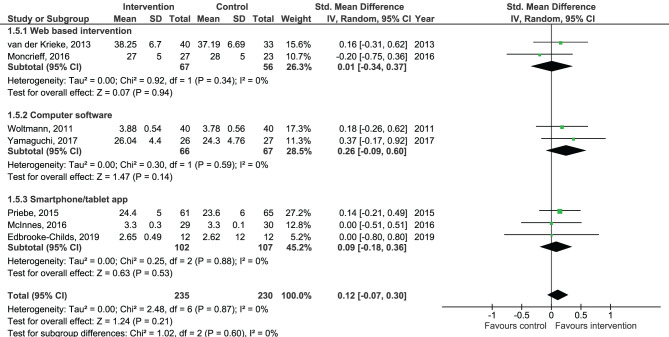
Forest plot for patient satisfaction by type of intervention.

**Figure 8:**
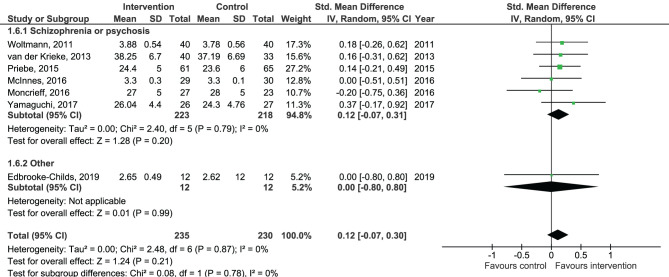
Forest plot for patient satisfaction by diagnosis.

**Figure 9:**
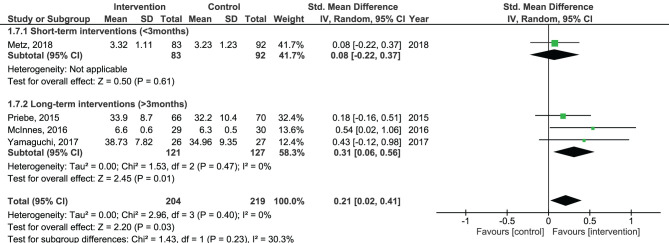
Forest plot for working alliance by duration perceived by the patient.

**Figure 10:**
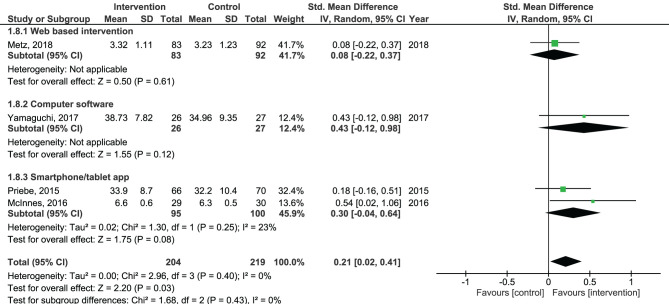
Forest plot for working alliance by type of intervention perceived by the patient.

**Figure 11:**
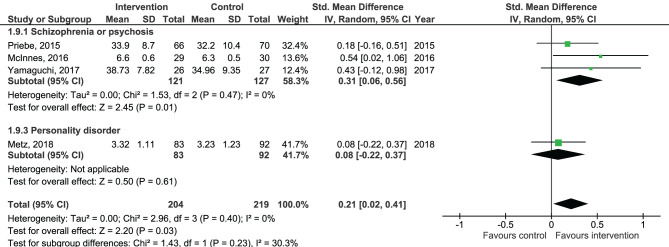
Forest plot for working alliance by diagnosis perceived by the patient.

**Figure 12:**
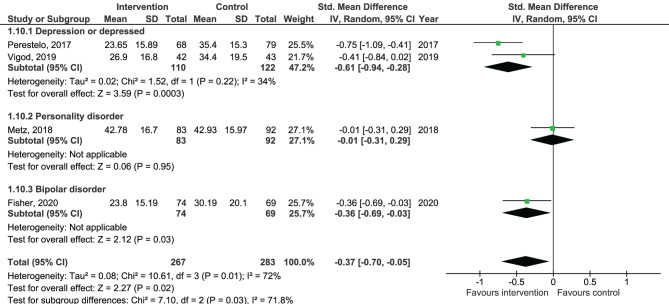
Forest plot for decisional conflict by diagnosis.

Results of the pooled effect size from digital SDM interventions on the secondary outcomes are presented in Figures 6–12. The random-effects meta-analysis revealed a small positive effect of digital SDM interventions for improving working alliance (four studies, *N* = 423, SMD = 0.21, CI: 0.02, 0.41, *p* = 0.03) (16, 28, 30, 31), a small-to-moderate positive effect for improving decisional conflict (four studies, *N* = 550, SMD = −0.37, CI: −0.70, −0.05, *p* = 0.02) (15–18), and no significant effect on patient satisfaction (seven studies, *N* = 465, SMD = 0.12, CI: −0.07, 0.30, *p* = 0.21) (21, 22, 24, 27, 28, 30, 31). Variation across trials due to heterogeneity was present for decisional conflict (Chi^2^ = 10.69, *p* = 0.01, *I*^2^ = 72%) but not for satisfaction (Chi^2^ = 2.48, *p* = 0.87, *I*^2^ = 0%) and working alliance (Chi^2^ = 2.96, *p* = 0.04, *I*^2^ = 0%). Assessment of publication bias was only performed for patient satisfaction due to the limited amount of studies for each pooled outcome. Via visual inspection of funnel plots, we did not assess any publication bias for patient satisfaction. Subgroup analysis on satisfaction by duration, type of intervention, and diagnosis showed no statistical significant differences between groups (Figures 6–8). Subgroup analysis on working alliance by duration, type of intervention, and diagnosis showed no statistical significant differences between groups (Figures 9–11). Subgroup analysis on decisional conflict by diagnosis showed a tendency for a greater effect for populations with symptoms of depression than other types of symptoms (two studies, *N* = 232, SMD = −0.61 CI: −0.94, −0.28, *p* = 0.0003; Figure 12). Subgroup analysis on decisional conflict by duration and type of intervention was not performed due to all studies being in the same subgroup.

### Assessment of Quality

The outcomes had a GRADE rating of very low quality for patient activation and low quality for self-efficacy, adverse events, symptoms, working alliance, decisional conflict, and patient satisfaction. This implies that we have little confidence in the estimates of effect for these outcomes.

### Narrative Synthesis of Intervention Effects

Table 2 presents the results of the narrative synthesis of 39 outcomes. The results on these outcomes were mixed between showing no difference between the groups and favoring the intervention group. None of the studies found effects favoring the control group.

**Table 2:** Results of the narrative synthesis of 39 outcomes.

**Outcomes**	**Number of trials favoring intervention group**	**Number of trials showing no difference between groups**	**Number of trials favoring control group**
**Objectively reported**			
Visit duration	1 (14)		
Patient contribution	1 (14)		
Reduce amount of clinician talk	1 (14)		
Amount of questions asked	1 (14)		
Patient-Centered Communication		1 (29)	
Receiving an antidepressant recommendation or mental health referral	1 (25)		
Did patient ask provider for information	1 (25)		
Social functioning		1 (28)	
Weight		1 (26)	
Level of functioning		1 (26)	
**Subjectively reported**			
Patient perceived involvement		1 (18)	
Knowledge	1 (15, 16, 27)	1 (17)	
Preference for participation in decision making		1 (15)	
Attitude toward medication	1 (24)		
Unmet needs	2 (28, 30)		
Provider satisfaction	1 (16)		
Empowerment (not compatible with review manager)		1 (21)	
Self-Determination		1 (21)	
Recovery	1 (21)	2 (26, 31)	
Patient-Centered communication perceived by patient		1 (29)	
Patient-Centered communication perceived by provider		1 (29)	
Level of shared decision-making	2 (20, 26)	1 (22)	
Strengths and difficulties		1 (22)	
Quality of life	1 (30)	3 (26, 28, 31)	
Level of burnout (provider)		1 (31)	
Institution's social atmosphere		1 (31)	
Overall mental well-being		2 (25, 28)	
Overall physical well-being		1 (25)	
Working alliance (provider perspective)	1 (26)	1 (28)	
Understanding of treatment options	1 (27)		
Feeling prepared for decision making	1 (27)		
Decisional regret	1 (27)		
Quality of communication with provider	1 (26)		
Medication adherence	1 (24)	1 (26)	
Treatment intention	1 (15)		
Treatment adherence (no-show, drop-out)		1 (20)	
Satisfaction (not compatible with review manager)	1 (30)	1 (16)	
Disturbed behavior	1 (31)		
Anxiety (Trait)		1 (17)	

*While we planned to perform meta-analyses on adherence to treatment and hospital admissions, these analyses were not applicable since <2 trials reported data on these aspects. Analysis on level of functioning was not performed due to the scales not being comparable. Analysis on adherence/usage of medicine was not performed due to only two studies reporting this area with one of the studies reporting data not compatible with review manager. The outcomes were therefore included in the narrative synthesis*.

## Discussion

Our review on the effectiveness of digital SDM interventions in mental healthcare included 2,400 participants across 16 RCTs examining digital interventions to support SDM with the majority conducted on psychosis, schizophrenia, or similar disorders.

The main analysis found that digital SDM interventions led to a moderate significant effect on improving patient activation in mental healthcare but not on self-efficacy, empowerment, or subjective level of patient involvement. Such result could be that digital SDM tools are more effective at addressing the concepts of patient activation. However, only two small sampled studies investigated the effectiveness on patient activation, three studies on self-efficacy, and one study each on empowerment and patient-involvement. Therefore, more quality research on the effectiveness of SDM interventions on patient activation or indices of the same are greatly needed to investigate this further.

As for symptoms, the use of a digital SDM intervention had a significant effect on general symptoms but not on positive, negative, anxiety, depressive, or overall psychiatric symptoms. Still, subgroup analysis showed no differences between types of symptoms, and with the GRADE level of the symptom outcomes, more research is needed to assess the effects of digital SDM tools on severity of symptoms.

We identified two trials that included an outcome of adverse events showing no significant differences between groups. In addition, none of the studies investigated potential negative effects of using the digital intervention. The current evidence on the effectiveness of digital interventions on adverse events is scarce, which also has been highlighted by other systematic reviews (32). While there are many positive possibilities with digital health technology, it is essential to also examine the potential negative effects of these tools especially with the continuous and rapid development of IT.

For the secondary outcomes, a small significant effect was found for working alliance and decisional conflict. The most frequent outcome measured by the 16 trials, patient satisfaction, revealed no significant effect; but authors of the included trials indicates that “instruments focusing on satisfaction might suffer from ceiling effects” (21). As previous reviews have reported mixed results on patient satisfaction (9, 33) with it being advocated as an argument for implementing SDM (4), future trials may wish to consider their choice of measurement for patient satisfaction and pilot test to identify a potential ceiling effect. Lastly, the narrative synthesis indicates a broad range of outcomes that digital SDM interventions may have an effect on, such as knowledge, unmet needs, and the level of SDM.

The significant effect on patient activation, working alliance, and decisional conflict may indicate that SDM benefits the collaboration between patient and provider. Future research may wish to investigate whether SDM is directly associated with an effect on health outcomes or if the collaboration serves as a mediator for health outcomes (e.g., severity of symptoms).

The fact that the most frequent outcome measures in this review were assessed by half of the trials and that only two trials measured our planned primary outcome, patient activation, highlights the vast differences in how trials evaluate their SDM interventions. Furthermore, although all included interventions support SDM, only three studies directly measured the level of SDM (16, 27, 31). According to our protocol, we had also planned to conduct meta-analyses on other outcomes (i.e., adherence to treatment, hospital admissions, level of functioning, and adherence/usage of medicine) (11). These analyses were, however, not possible due to either lack of studies assessing the outcome or outcomes not being compatible for meta-analysis. These outcomes were instead included in the narrative synthesis. The vast differences in how studies evaluate their SDM intervention create a limitation for reviews and meta-analysis since the combined data for each specific outcome are scarce.

Although all included studies investigated an intervention to support SDM, none of the included studies addressed all aspects of SDM, and only three studies included a measurement to assess the overall level of SDM. This may highlight a challenge in how to measure and evaluate the effect on SDM. The included trials in this review could be divided into two groups of systems: (1) a system developed to be used independently of the involvement of a provider or (2) a system developed to be incorporated into the collaboration with the provider. A system to support the patient could be a tool aimed at improving the patient's knowledge on needs, values, options, and the feeling of being prepared for the consultation. A system to support the consultation could be a tool aimed to ensure that a range of life domains and treatment aspects were consistently and structurally addressed and that patients' perspectives were always elicited at the consultation. For interventions not actively including the provider, patients were still encouraged to use the tool in collaboration with their provider (e.g., sharing one's self-assessments or showing one's notes). However, a challenge occurs when either the patient or provider is not actively included: the responsibility of incorporating and using the tool in the consultation is no longer shared and is instead solely placed on either the patient or provider—a responsibility that may be overwhelming for some. Also, as reported by one of the studies “A one-off intervention […] may be insufficient to improve patient involvement in decision-making” (22), indicating that tools to support patients may be helpful but insufficient on their own. Similarly, SDM on its own has shown to be difficult to incorporate into clinical practice due to its complexity and vagueness on how to translate its theoretical model into practice. Therefore, research has called for diverse ways in which SDM principles can be translated into practice such as decision aids (33).

With the vast differences in how researchers are developing tools to support SDM, quality guidance to develop and to assess these tools are needed. Such assessment is possible for, e.g., patient decision aids where International Patient Decision Aid Standards (IPDAS) may be used to assess the quality of the tool (18). In this review, five studies defined their intervention as a decision aid, but only one study mentioned having developed their intervention based on IPDAS guidelines. Since decision aids are not the only mean to support SDM, similar assessment tools could assist in providing clarity on the similarities between tools to support SDM and their level of quality. In addition, future trials investigating the effect of a digital SDM intervention are encouraged to consider including a measurement of adherence/usage of the tool and have their participants evaluate the tool. Reporting an observed effect of a tool in combination with data on how the tool was used will assist future trials and stakeholders to determine whether an effect is dependent on a certain level of usage or acceptance or if the participants found the tool meaningful. While digital interventions may be able to address some of the barriers associated with SDM, it is also important to consider what barriers are introduced with a digital intervention. Traditional barriers for digital interventions may be privacy and data security concerns, but there is also a need for more evidence on how digital interventions may be influenced by variables such as user engagement, data-driven feedback, or individual expectations and characteristics (34).

A majority of the included trials investigated either a web-based intervention or a computer software, while only three studies investigated a smartphone or tablet application. This limited the possibilities to assess the effectiveness of a digital intervention depending on its features or system category (e.g., web-based, computer software, and smartphone app). To determine what digital features are the most effective at supporting SDM, more research investigating different types of features is needed.

### Strengths and Limitations

Our review has several strengths. Firstly, it provides evidence regarding the effectiveness of digital SDM interventions, which has not been conducted before. Secondly, this meta-analysis strictly follows the registered protocol describing our search strategy, types of studies to be included, data extraction, and targeted outcome measures (11). The only change was the inclusion of one extra secondary outcome, which was done due to several studies assessing this outcome (decisional conflict). A limitation to the study was our inclusion criteria on SDM. The complexness of SDM creates several ways to support SDM, which may cause high heterogeneity between studies. Because of the broad definition of SDM, many of the included trials share similarities while also differing from one another. Subgroup analysis on what components of SDM were used could be highly relevant to identify what aspect of SDM is providing the largest effectiveness. However, such subgroup analysis may be difficult without a clear definition of the SDM process and necessitates that studies clearly describe how their intervention supports SDM. Our study was also limited by not acquiring unpublished literature and assessing publication bias for only one outcome due to the limited amount of studies reporting the same outcomes.

## Conclusions

Digital interventions to support SDM may be a promising tool in mental healthcare. The complexness of SDM and possibilities with digital tools create many possibilities for researchers as showcased in this review. It is still unclear which features of digital tools are most effective at supporting the SDM process. More quality research is needed to further assess the effectiveness of digital means to support SDM but also to determine which intervention features are most effective in supporting SDM.

## Data Availability Statement

The data analyzed in this study is subject to the following licenses/restrictions: Most data from the included trials can be found online. For some trials, we had to contact the corresponding author to receive the data for analysis. Requests to access these datasets should be directed to tobias.vitger@regionh.dk (I am happy to put you in contact with those authors who delivered raw data).

## Author Contributions

TV, CH, and LK designed the search strategy and the study protocol. TV and CH screened the identified studies against inclusion/exclusion criteria to determine eligibility for selection. TV performed the data analysis under supervision of CH and wrote the first draft of the manuscript. CH, LK, MN, SA, and LP all critically revised the manuscript. All authors approved the final version of the manuscript.

## Conflict of Interest

The authors declare that the research was conducted in the absence of any commercial or financial relationships that could be construed as a potential conflict of interest.

## Publisher's Note

All claims expressed in this article are solely those of the authors and do not necessarily represent those of their affiliated organizations, or those of the publisher, the editors and the reviewers. Any product that may be evaluated in this article, or claim that may be made by its manufacturer, is not guaranteed or endorsed by the publisher.
