# Supplementary figures and images for: Digital Shared Decision-Making Interventions in Mental Healthcare: A Systematic Review and Meta-Analysis
Source: Front Psychiatry. 2021 Sep 6;12:691251. doi: 10.3389/fpsyt.2021.691251 (PMC8450495; doi:10.3389/fpsyt.2021.691251)

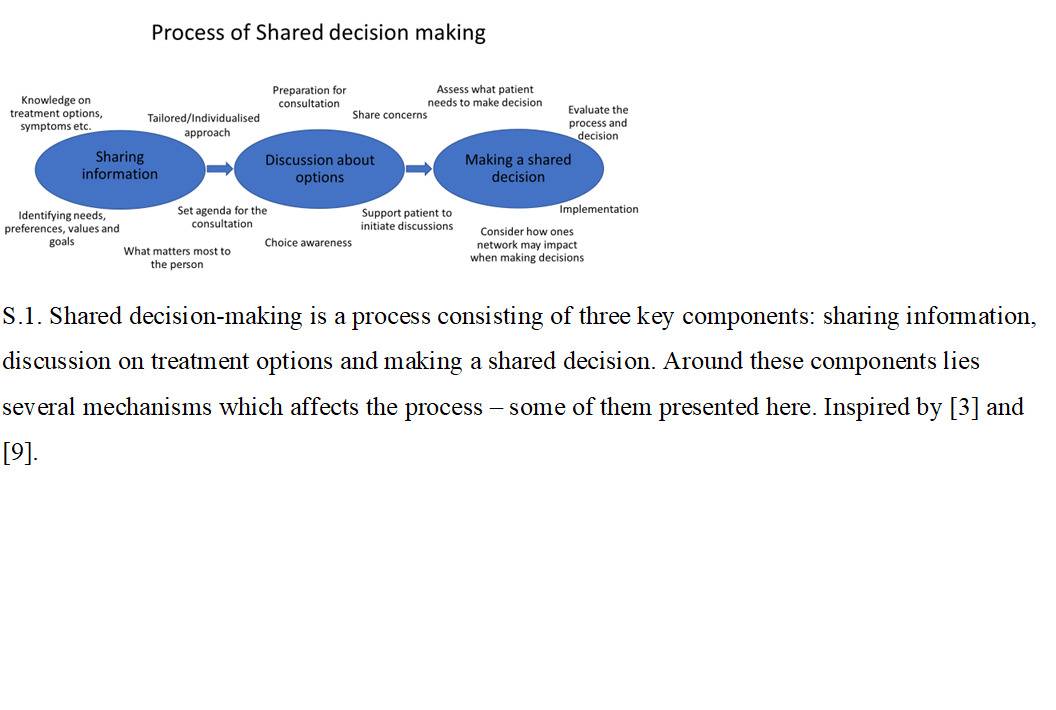

Supplement: Supplementary file 1 [file Image_1.JPEG]
