# Supplementary material for: Digital Shared Decision-Making Interventions in Mental Healthcare: A Systematic Review and Meta-Analysis
Source: Front Psychiatry. 2021 Sep 6;12:691251. doi: 10.3389/fpsyt.2021.691251 (PMC8450495; doi:10.3389/fpsyt.2021.691251)
Supplement: Supplementary file 2 [file Data_Sheet_2.pdf]

Study or Subgroup	Intervention Mean	Intervention SD	Intervention Total	Control Mean	Control SD	Control Total	Weight	Std. Mean Difference IV, Random, 95% CI
**1.1.1 Patient activation Long-term intervention**								
Yamaguchi, 2017	52.28	18.13	26	45.78	10.82	27	9.6%	0.43 [-0.11, 0.98]
**Subtotal (95% CI)**			**26**			**27**	**9.6%**	**0.43 [-0.11, 0.98]**
Heterogeneity: Not applicable								
Test for overall effect: Z = 1.55 (P = 0.12)								
**1.1.2 Patient involvement Short-term interventions**								
Metz, 2018	34.53	10.82	83	34.43	10.01	92	20.6%	0.01 [-0.29, 0.31]
**Subtotal (95% CI)**			**83**			**92**	**20.6%**	**0.01 [-0.29, 0.31]**
Heterogeneity: Not applicable								
Test for overall effect: Z = 0.06 (P = 0.95)								
**1.1.3 Patient activation Short-term intervention**								
Edbrooke-Childs, 2019	40.34	5.43	12	35.59	5.31	12	4.7%	0.85 [0.01, 1.70]
**Subtotal (95% CI)**			**12**			**12**	**4.7%**	**0.85 [0.01, 1.70]**
Heterogeneity: Not applicable								
Test for overall effect: Z = 1.99 (P = 0.05)								
**1.1.6 Self-efficacy Long-term intervention**								
Priebe, 2015	26.5	6.6	73	26.5	6.2	73	18.8%	0.00 [-0.32, 0.32]
**Subtotal (95% CI)**			**73**			**73**	**18.8%**	**0.00 [-0.32, 0.32]**
Heterogeneity: Not applicable								
Test for overall effect: Z = 0.00 (P = 1.00)								
**1.1.7 Self-efficacy Short-term intervention**								
Kravitz, 2015	22.94	6.69	294	23.01	6.32	287	31.1%	-0.01 [-0.17, 0.15]
Moncrieff, 2016	30	9	31	32	10	29	10.6%	-0.21 [-0.72, 0.30]
**Subtotal (95% CI)**			**325**			**316**	**41.7%**	**-0.03 [-0.18, 0.13]**
Heterogeneity: Tau² = 0.00; Chi² = 0.53, df = 1 (P = 0.47); I² = 0%								
Test for overall effect: Z = 0.37 (P = 0.71)								
**1.1.8 empowerment Short-term intervention**								
Edbrooke-Childs, 2019	22.33	3.31	12	19.18	4.17	12	4.7%	0.81 [-0.03, 1.65]
**Subtotal (95% CI)**			**12**			**12**	**4.7%**	**0.81 [-0.03, 1.65]**
Heterogeneity: Not applicable								
Test for overall effect: Z = 1.89 (P = 0.06)								

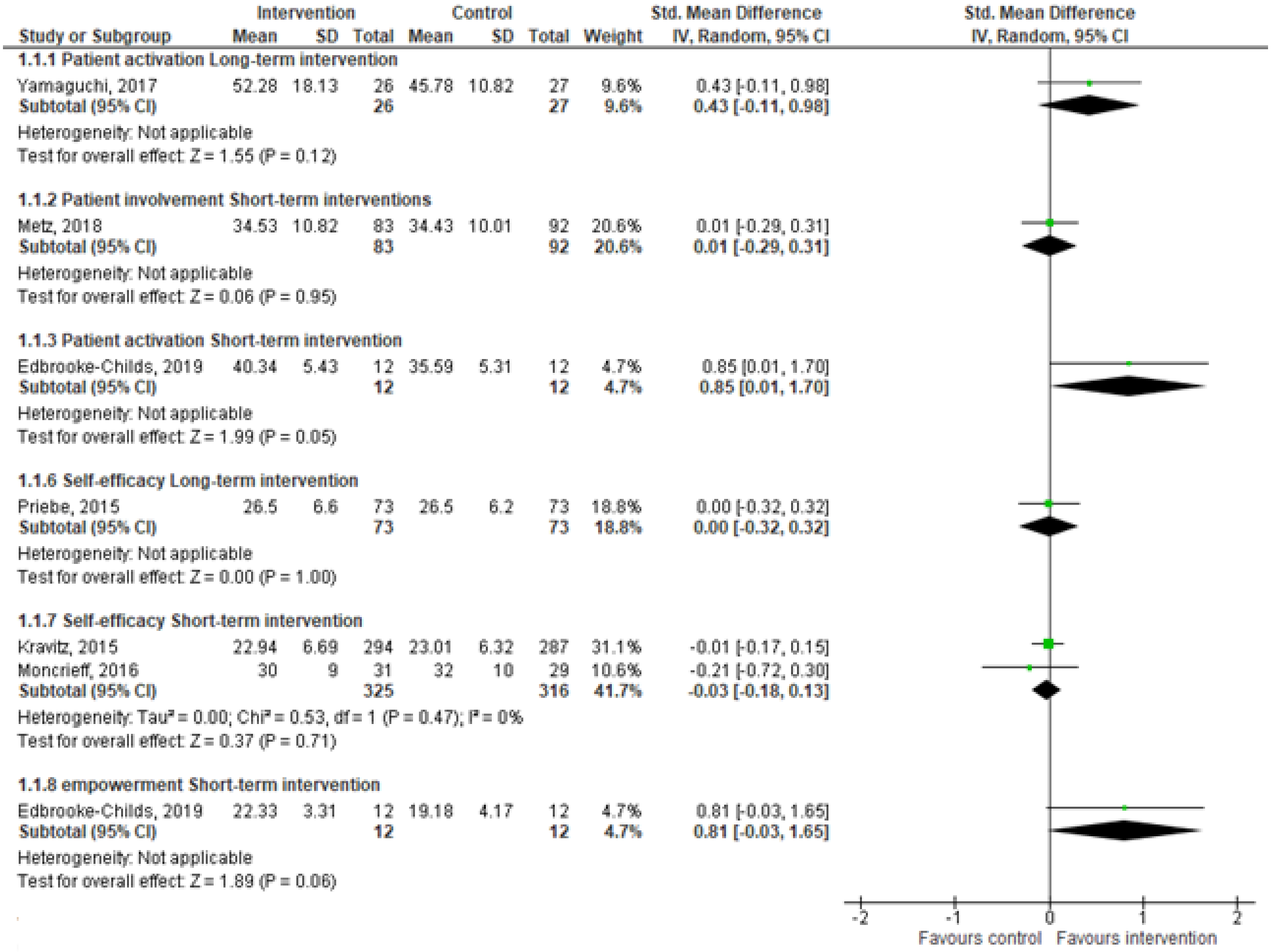

S.3. Forest plot on patient activation, self-efficacy and empowerment by duration.

	Intervention			Control				Std. Mean Difference
Study or Subgroup	Mean	SD	Total	Mean	SD	Total	Weight	IV, Random, 95% CI
**1.1.2 Patient activation Computer software**								
Yamaguchi, 2017	52.28	18.13	26	45.78	10.82	27	9.6%	0.43 [-0.11, 0.98]
**Subtotal (95% CI)**			**26**			**27**	**9.6%**	**0.43 [-0.11, 0.98]**
Heterogeneity: Not applicable								
Test for overall effect: Z = 1.55 (P = 0.12)								
**1.1.3 Patient activation Smartphone/tablet app**								
Edbrooke-Childs, 2019	40.34	5.43	12	35.59	5.31	12	4.7%	0.85 [0.01, 1.70]
**Subtotal (95% CI)**			**12**			**12**	**4.7%**	**0.85 [0.01, 1.70]**
Heterogeneity: Not applicable								
Test for overall effect: Z = 1.99 (P = 0.05)								
**1.1.4 Patient involvement Web based intervention**								
Metz, 2018	34.53	10.82	83	34.43	10.01	92	20.6%	0.01 [-0.29, 0.31]
**Subtotal (95% CI)**			**83**			**92**	**20.6%**	**0.01 [-0.29, 0.31]**
Heterogeneity: Not applicable								
Test for overall effect: Z = 0.06 (P = 0.95)								
**1.1.6 Self-efficacy Web based intervention**								
Moncrieff, 2016	30	9	31	32	10	29	10.6%	-0.21 [-0.72, 0.30]
**Subtotal (95% CI)**			**31**			**29**	**10.6%**	**-0.21 [-0.72, 0.30]**
Heterogeneity: Not applicable								
Test for overall effect: Z = 0.80 (P = 0.42)								
**1.1.7 Self-efficacy Computer software**								
Kravitz, 2015	22.94	6.69	294	23.01	6.32	287	31.1%	-0.01 [-0.17, 0.15]
**Subtotal (95% CI)**			**294**			**287**	**31.1%**	**-0.01 [-0.17, 0.15]**
Heterogeneity: Not applicable								
Test for overall effect: Z = 0.13 (P = 0.90)								
**1.1.8 Self-efficacy smartphone/tablet app**								
Priebe, 2015	26.5	6.6	73	26.5	6.2	73	18.8%	0.00 [-0.32, 0.32]
**Subtotal (95% CI)**			**73**			**73**	**18.8%**	**0.00 [-0.32, 0.32]**
Heterogeneity: Not applicable								
Test for overall effect: Z = 0.00 (P = 1.00)								
**1.1.9 empowerment smartphone/tablet app**								
Edbrooke-Childs, 2019	22.33	3.31	12	19.18	4.17	12	4.7%	0.81 [-0.03, 1.65]
**Subtotal (95% CI)**			**12**			**12**	**4.7%**	**0.81 [-0.03, 1.65]**
Heterogeneity: Not applicable								
Test for overall effect: Z = 1.89 (P = 0.06)								

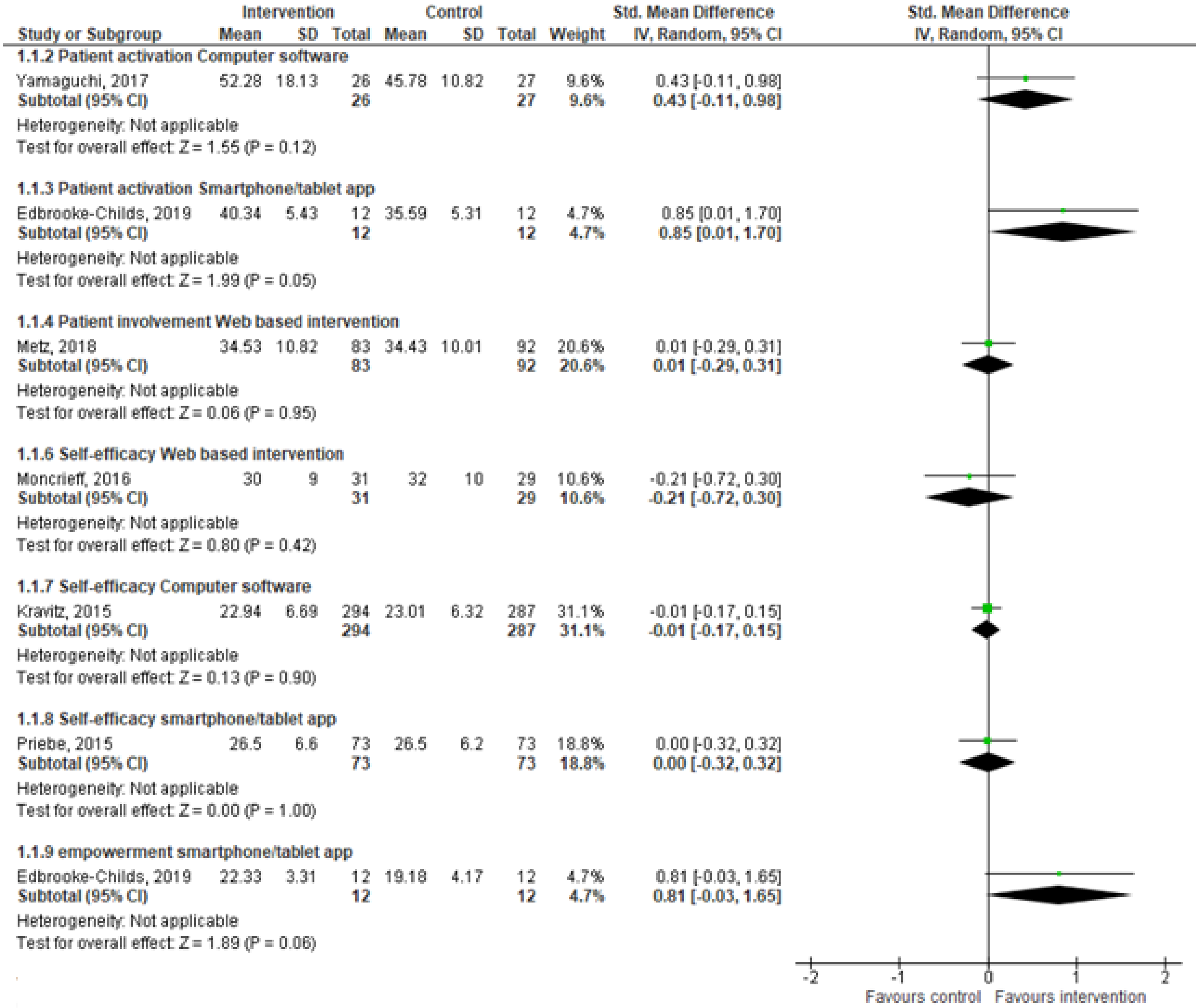

S.4. Forest plot on patient activation, self-efficacy and empowerment by type of intervention.

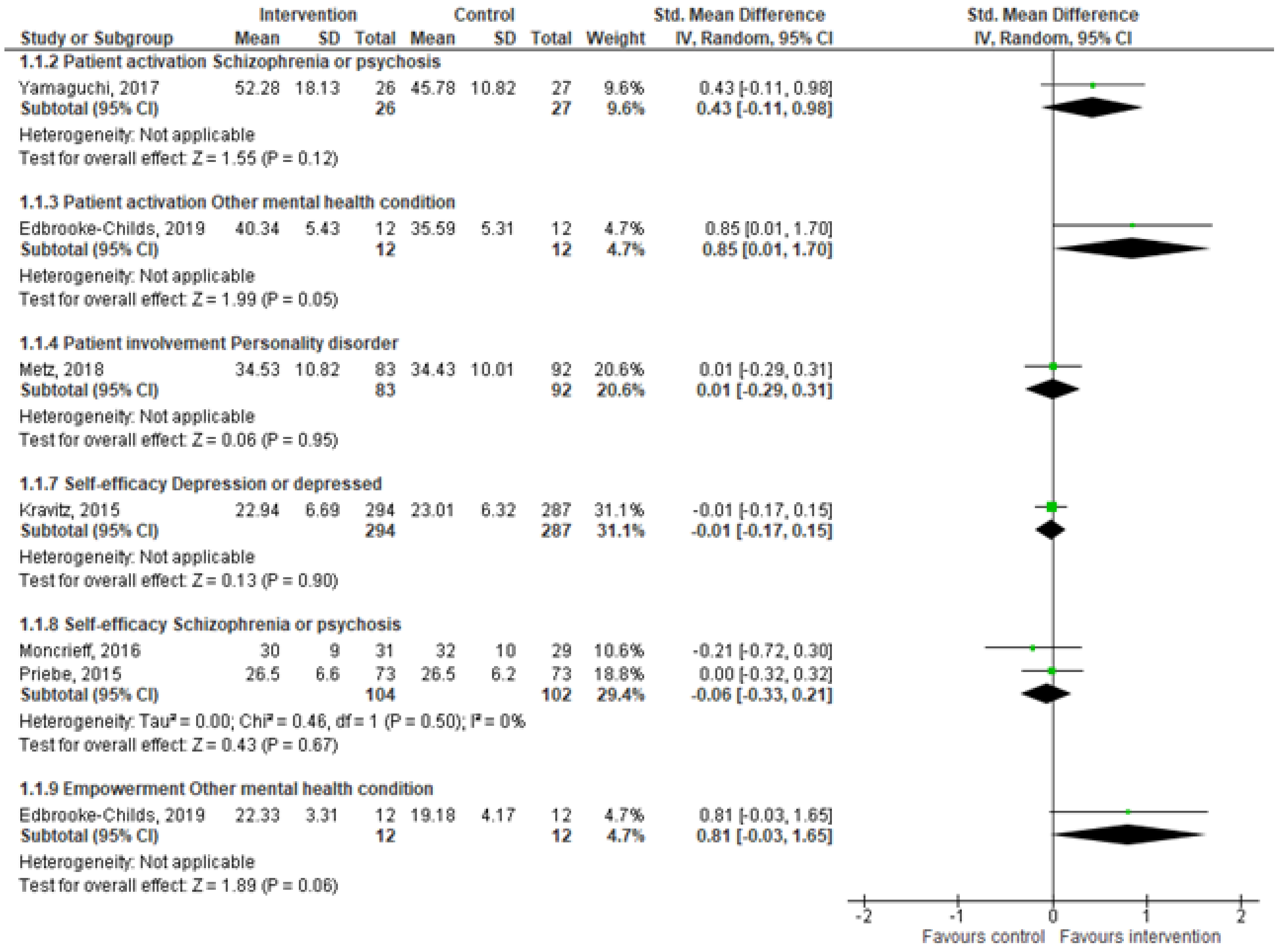

S.5. Forest plot on patient activation, self-efficacy and empowerment by diagnosis.

Study or Subgroup	Intervention Mean	Intervention SD	Intervention Total	Control Mean	Control SD	Control Total	Weight	Std. Mean Difference IV, Fixed, 95% CI	Year
**1.15.1 Positive symptoms Long term intervention (>3months)**									
Priebe, 2007	14.07	5.9	241	14.82	5.88	206	17.0%	-0.13 [-0.31, 0.06]	2007
Priebe, 2015	13.2	5.2	73	14.4	5.7	73	5.6%	-0.22 [-0.54, 0.11]	2015
**Subtotal (95% CI)**			**314**			**279**	**22.6%**	**-0.15 [-0.31, 0.01]**	
Heterogeneity: Chi^2 = 0.23, df = 1 (P = 0.63); I^2 = 0%									
Test for overall effect: Z = 1.82 (P = 0.07)									
**1.15.2 Negative symptoms Long term intervention (>3months)**									
Priebe, 2007	15.16	6.51	240	15.61	6.35	208	17.1%	-0.07 [-0.26, 0.12]	2007
Priebe, 2015	15.1	5.8	73	15.7	6.1	73	5.6%	-0.10 [-0.42, 0.22]	2015
**Subtotal (95% CI)**			**313**			**281**	**22.7%**	**-0.08 [-0.24, 0.08]**	
Heterogeneity: Chi^2 = 0.03, df = 1 (P = 0.87); I^2 = 0%									
Test for overall effect: Z = 0.94 (P = 0.35)									
**1.15.3 General symptoms Long term intervention (>3months)**									
Priebe, 2007	30.48	9.78	241	30.94	9.5	207	17.1%	-0.05 [-0.23, 0.14]	2007
Priebe, 2015	28	9.2	73	32.8	8.9	73	5.4%	-0.53 [-0.86, -0.20]	2015
**Subtotal (95% CI)**			**314**			**280**	**22.5%**	**-0.16 [-0.32, -0.00]**	
Heterogeneity: Chi^2 = 6.17, df = 1 (P = 0.01); I^2 = 84%									
Test for overall effect: Z = 1.97 (P = 0.05)									
**1.15.4 General symptoms Short term intervention (<3months)**									
Metz, 2018	64.18	15.27	83	67.6	18.45	92	6.7%	-0.20 [-0.50, 0.10]	2018
**Subtotal (95% CI)**			**83**			**92**	**6.7%**	**-0.20 [-0.50, 0.10]**	
Heterogeneity: Not applicable									
Test for overall effect: Z = 1.32 (P = 0.19)									
**1.15.5 Overall psychiatric symptoms Long term intervention (>3months)**									
Yamaguchi, 2017	33.96	7.85	26	35.41	6.95	27	2.0%	-0.19 [-0.73, 0.35]	2017
**Subtotal (95% CI)**			**26**			**27**	**2.0%**	**-0.19 [-0.73, 0.35]**	
Heterogeneity: Not applicable									
Test for overall effect: Z = 0.70 (P = 0.48)									
**1.15.6 Overall psychiatric symptoms Short term intervention (<3months)**									
Moncrieff, 2016	16	6	27	16	7	23	1.9%	0.00 [-0.56, 0.56]	2016
**Subtotal (95% CI)**			**27**			**23**	**1.9%**	**0.00 [-0.56, 0.56]**	
Heterogeneity: Not applicable									
Test for overall effect: Z = 0.00 (P = 1.00)									
**1.15.7 Anxiety Short term intervention (<3months)**									
Vigod, 2019	39.8	14.5	41	42.2	13.3	43	3.2%	-0.17 [-0.60, 0.26]	2019
Fisher, 2020	45.44	15.12	38	51.21	15.65	44	3.1%	-0.37 [-0.81, 0.07]	2020
**Subtotal (95% CI)**			**79**			**87**	**6.3%**	**-0.27 [-0.58, 0.04]**	
Heterogeneity: Chi^2 = 0.41, df = 1 (P = 0.52); I^2 = 0%									
Test for overall effect: Z = 1.72 (P = 0.09)									
**1.15.8 Depressive symptoms Short term intervention (<3months)**									
Kravitz, 2015	8.6	5.29	168	7.6	5.02	151	12.2%	0.19 [-0.03, 0.41]	2015
Vigod, 2019	9	5.15	41	10.3	4.92	43	3.2%	-0.26 [-0.69, 0.17]	2019
**Subtotal (95% CI)**			**209**			**194**	**15.3%**	**0.10 [-0.10, 0.30]**	
Heterogeneity: Chi^2 = 3.32, df = 1 (P = 0.07); I^2 = 70%									
Test for overall effect: Z = 1.00 (P = 0.32)									

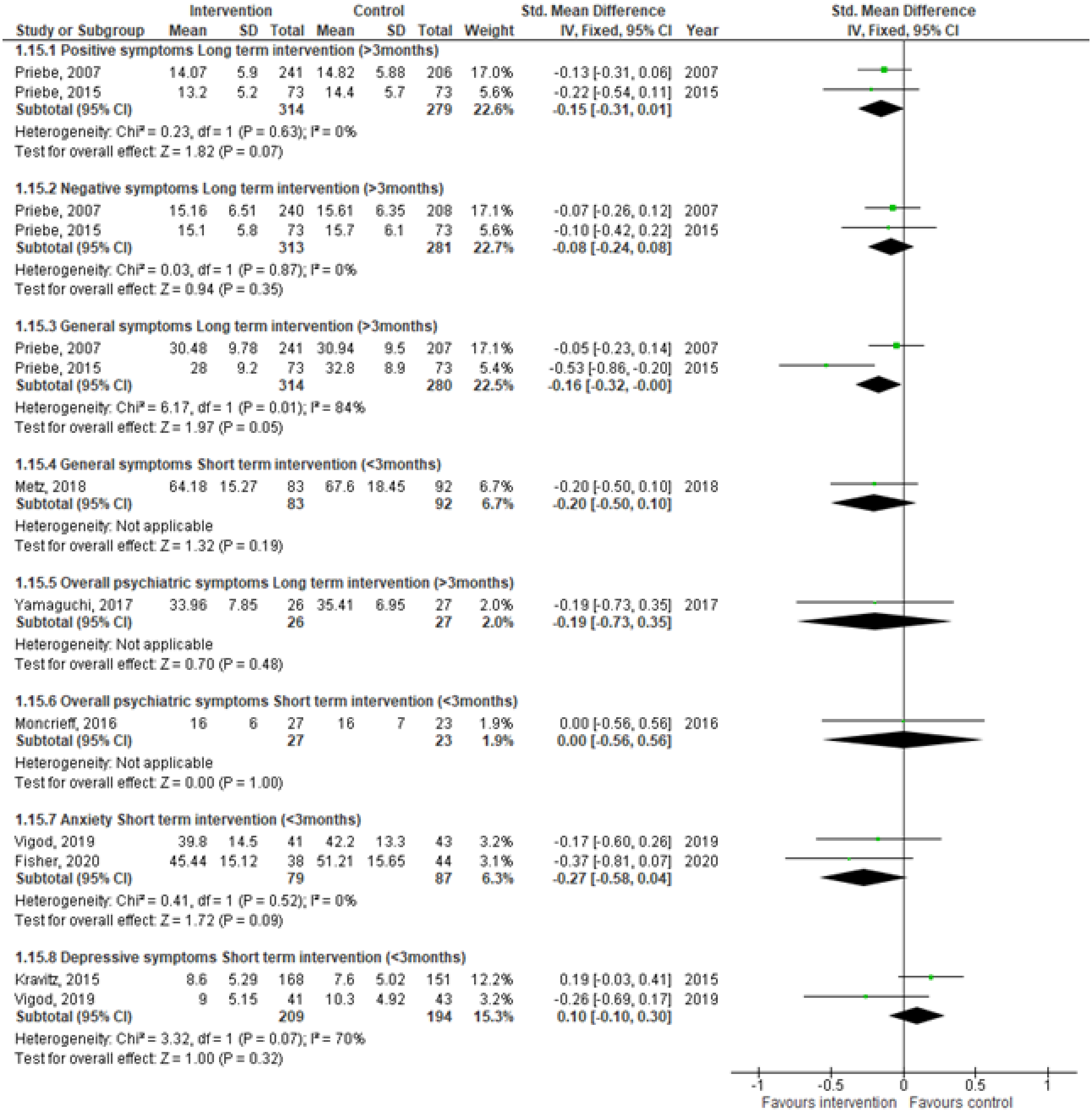

S.6. Forest plot on symptoms by duration.

Study or Subgroup	Intervention Mean	Intervention SD	Intervention Total	Control Mean	Control SD	Control Total	Weight	Std. Mean Difference IV, Fixed, 95% CI	Year
**1.16.1 Positive symptoms Computer software**									
Priebe, 2007	14.07	5.9	241	14.82	5.88	206	17.0%	-0.13 [-0.31, 0.06]	2007
**Subtotal (95% CI)**			**241**			**206**	**17.0%**	**-0.13 [-0.31, 0.06]**	
Heterogeneity: Not applicable									
Test for overall effect: Z = 1.34 (P = 0.18)									
**1.16.2 Positive symptoms Smartphone/tablet app**									
Priebe, 2015	13.2	5.2	73	14.4	5.7	73	5.6%	-0.22 [-0.54, 0.11]	2015
**Subtotal (95% CI)**			**73**			**73**	**5.6%**	**-0.22 [-0.54, 0.11]**	
Heterogeneity: Not applicable									
Test for overall effect: Z = 1.32 (P = 0.19)									
**1.16.3 Negative symptoms Computer software**									
Priebe, 2007	15.16	6.51	240	15.61	6.35	208	17.1%	-0.07 [-0.26, 0.12]	2007
**Subtotal (95% CI)**			**240**			**208**	**17.1%**	**-0.07 [-0.26, 0.12]**	
Heterogeneity: Not applicable									
Test for overall effect: Z = 0.74 (P = 0.46)									
**1.16.4 Negative symptoms Smartphone/tablet app**									
Priebe, 2015	15.1	5.8	73	15.7	6.1	73	5.6%	-0.10 [-0.42, 0.22]	2015
**Subtotal (95% CI)**			**73**			**73**	**5.6%**	**-0.10 [-0.42, 0.22]**	
Heterogeneity: Not applicable									
Test for overall effect: Z = 0.61 (P = 0.54)									
**1.16.5 General symptoms Computer software**									
Priebe, 2007	30.48	9.78	241	30.94	9.5	207	17.1%	-0.05 [-0.23, 0.14]	2007
**Subtotal (95% CI)**			**241**			**207**	**17.1%**	**-0.05 [-0.23, 0.14]**	
Heterogeneity: Not applicable									
Test for overall effect: Z = 0.50 (P = 0.62)									
**1.16.6 General symptoms Smartphone/tablet app**									
Priebe, 2015	28	9.2	73	32.8	8.9	73	5.4%	-0.53 [-0.86, -0.20]	2015
**Subtotal (95% CI)**			**73**			**73**	**5.4%**	**-0.53 [-0.86, -0.20]**	
Heterogeneity: Not applicable									
Test for overall effect: Z = 3.13 (P = 0.002)									
**1.16.7 General symptoms Web based intervention**									
Metz, 2018	64.18	15.27	83	67.6	18.45	92	6.7%	-0.20 [-0.50, 0.10]	2018
**Subtotal (95% CI)**			**83**			**92**	**6.7%**	**-0.20 [-0.50, 0.10]**	
Heterogeneity: Not applicable									
Test for overall effect: Z = 1.32 (P = 0.19)									
**1.16.8 Anxiety Web based intervention**									
Vigod, 2019	39.8	14.5	41	42.2	13.3	43	3.2%	-0.17 [-0.60, 0.26]	2019
Fisher, 2020	45.44	15.12	38	51.21	15.65	44	3.1%	-0.37 [-0.81, 0.07]	2020
**Subtotal (95% CI)**			**79**			**87**	**6.3%**	**-0.27 [-0.58, 0.04]**	
Heterogeneity: $Chi^2 = 0.41$, df = 1 (P = 0.52); $I^2 = 0\%$									
Test for overall effect: Z = 1.72 (P = 0.09)									
**1.16.9 Overall psychiatric symptoms Web based intervention**									
Moncrieff, 2016	16	6	27	16	7	23	1.9%	0.00 [-0.56, 0.56]	2016
**Subtotal (95% CI)**			**27**			**23**	**1.9%**	**0.00 [-0.56, 0.56]**	
Heterogeneity: Not applicable									
Test for overall effect: Z = 0.00 (P = 1.00)									
**1.16.10 Overall psychiatric symptoms Computer software**									
Yamaguchi, 2017	33.96	7.85	26	35.41	6.95	27	2.0%	-0.19 [-0.73, 0.35]	2017
**Subtotal (95% CI)**			**26**			**27**	**2.0%**	**-0.19 [-0.73, 0.35]**	
Heterogeneity: Not applicable									
Test for overall effect: Z = 0.70 (P = 0.48)									
**1.16.11 Depressive symptoms Web based intervention**									
Vigod, 2019	9	5.15	41	10.3	4.92	43	3.2%	-0.26 [-0.69, 0.17]	2019
**Subtotal (95% CI)**			**41**			**43**	**3.2%**	**-0.26 [-0.69, 0.17]**	
Heterogeneity: Not applicable									
Test for overall effect: Z = 1.17 (P = 0.24)									
**1.16.12 Depressive symptoms Computer software**									
Kravitz, 2015	8.6	5.29	168	7.6	5.02	151	12.2%	0.19 [-0.03, 0.41]	2015
**Subtotal (95% CI)**			**168**			**151**	**12.2%**	**0.19 [-0.03, 0.41]**	
Heterogeneity: Not applicable									
Test for overall effect: Z = 1.72 (P = 0.09)									

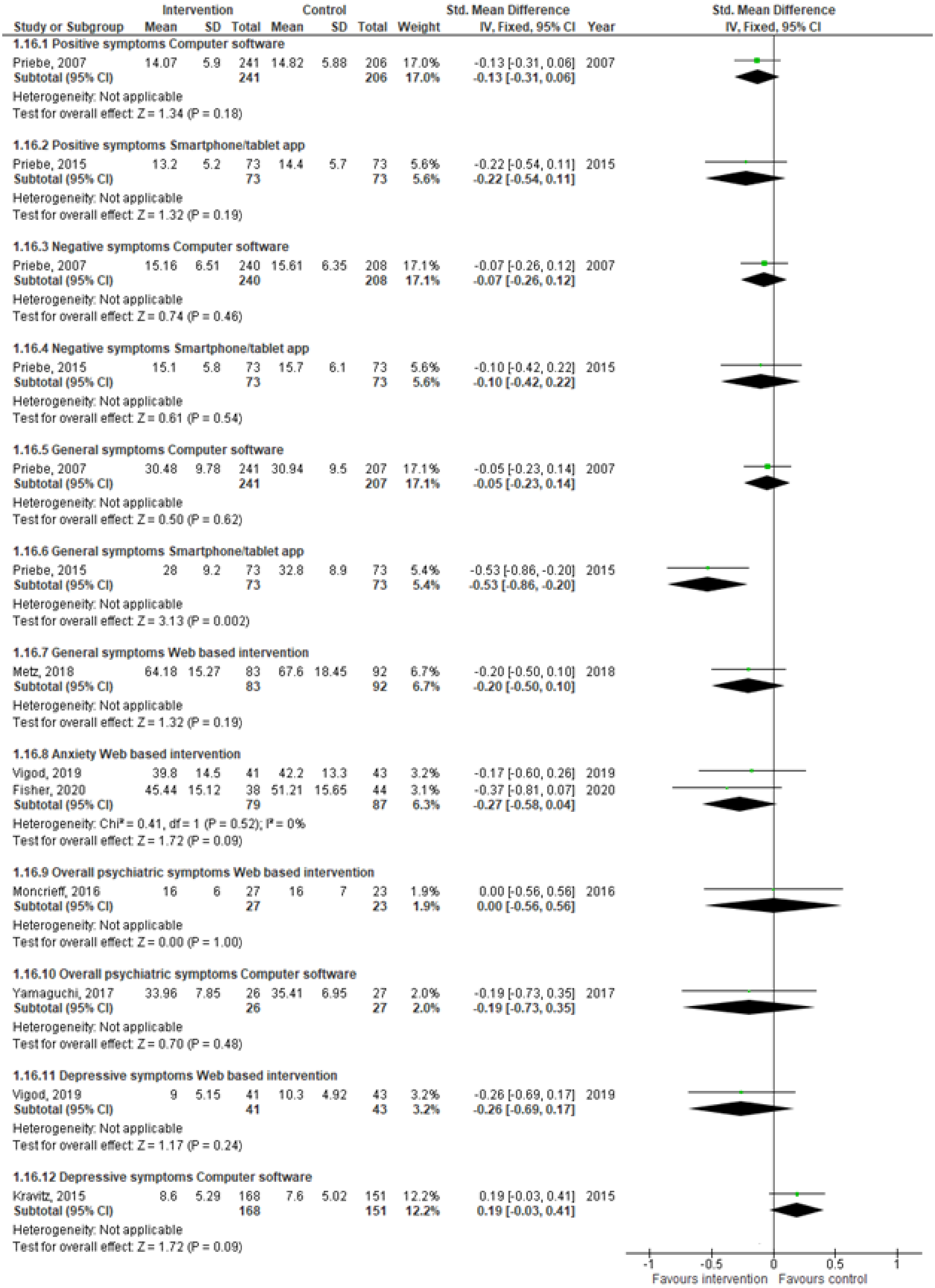

S.7. Forest plot on symptoms by type of intervention.

Study or Subgroup	Intervention Mean	Intervention SD	Intervention Total	Control Mean	Control SD	Control Total	Weight	Std. Mean Difference IV, Fixed, 95% CI	Year
**1.17.1 Positive symptoms Schizophrenia or psychosis**									
Priebe, 2007	14.07	5.9	241	14.82	5.88	206	17.0%	-0.13 [-0.31, 0.06]	2007
Priebe, 2015	13.2	5.2	73	14.4	5.7	73	5.6%	-0.22 [-0.54, 0.11]	2015
**Subtotal (95% CI)**			**314**			**279**	**22.6%**	**-0.15 [-0.31, 0.01]**	
Heterogeneity: Chi² = 0.23, df = 1 (P = 0.63); I² = 0%									
Test for overall effect: Z = 1.82 (P = 0.07)									
**1.17.2 Negative symptoms Schizophrenia or psychosis**									
Priebe, 2007	15.16	6.51	240	15.61	6.35	208	17.1%	-0.07 [-0.26, 0.12]	2007
Priebe, 2015	15.1	5.8	73	15.7	6.1	73	5.6%	-0.10 [-0.42, 0.22]	2015
**Subtotal (95% CI)**			**313**			**281**	**22.7%**	**-0.08 [-0.24, 0.08]**	
Heterogeneity: Chi² = 0.03, df = 1 (P = 0.87); I² = 0%									
Test for overall effect: Z = 0.94 (P = 0.35)									
**1.17.3 General symptoms Schizophrenia or psychosis**									
Priebe, 2007	30.48	9.78	241	30.94	9.5	207	17.1%	-0.05 [-0.23, 0.14]	2007
Priebe, 2015	28	9.2	73	32.8	8.9	73	5.4%	-0.53 [-0.86, -0.20]	2015
**Subtotal (95% CI)**			**314**			**280**	**22.5%**	**-0.16 [-0.32, -0.00]**	
Heterogeneity: Chi² = 6.17, df = 1 (P = 0.01); I² = 84%									
Test for overall effect: Z = 1.97 (P = 0.05)									
**1.17.4 General symptoms Personality disorder**									
Metz, 2018	64.18	15.27	83	67.6	18.45	92	6.7%	-0.20 [-0.50, 0.10]	2018
**Subtotal (95% CI)**			**83**			**92**	**6.7%**	**-0.20 [-0.50, 0.10]**	
Heterogeneity: Not applicable									
Test for overall effect: Z = 1.32 (P = 0.19)									
**1.17.5 Overall psychiatric symptoms Schizophrenia or psychosis**									
Moncrieff, 2016	16	6	27	16	7	23	1.9%	0.00 [-0.56, 0.56]	2016
Yamaguchi, 2017	33.96	7.85	26	35.41	6.95	27	2.0%	-0.19 [-0.73, 0.35]	2017
**Subtotal (95% CI)**			**53**			**50**	**3.9%**	**-0.10 [-0.49, 0.29]**	
Heterogeneity: Chi² = 0.24, df = 1 (P = 0.63); I² = 0%									
Test for overall effect: Z = 0.50 (P = 0.62)									
**1.17.6 Depressive symptoms Depression or depressed**									
Kravitz, 2015	8.6	5.29	168	7.6	5.02	151	12.2%	0.19 [-0.03, 0.41]	2015
Vigod, 2019	9	5.15	41	10.3	4.92	43	3.2%	-0.26 [-0.69, 0.17]	2019
**Subtotal (95% CI)**			**209**			**194**	**15.3%**	**0.10 [-0.10, 0.30]**	
Heterogeneity: Chi² = 3.32, df = 1 (P = 0.07); I² = 70%									
Test for overall effect: Z = 1.00 (P = 0.32)									
**1.17.7 Anxiety Depression or depressed**									
Vigod, 2019	39.8	14.5	41	42.2	13.3	43	3.2%	-0.17 [-0.60, 0.26]	2019
**Subtotal (95% CI)**			**41**			**43**	**3.2%**	**-0.17 [-0.60, 0.26]**	
Heterogeneity: Not applicable									
Test for overall effect: Z = 0.78 (P = 0.43)									
**1.17.8 Anxiety Bipolar disorder**									
Fisher, 2020	45.44	15.12	38	51.21	15.65	44	3.1%	-0.37 [-0.81, 0.07]	2020
**Subtotal (95% CI)**			**38**			**44**	**3.1%**	**-0.37 [-0.81, 0.07]**	
Heterogeneity: Not applicable									
Test for overall effect: Z = 1.66 (P = 0.10)									

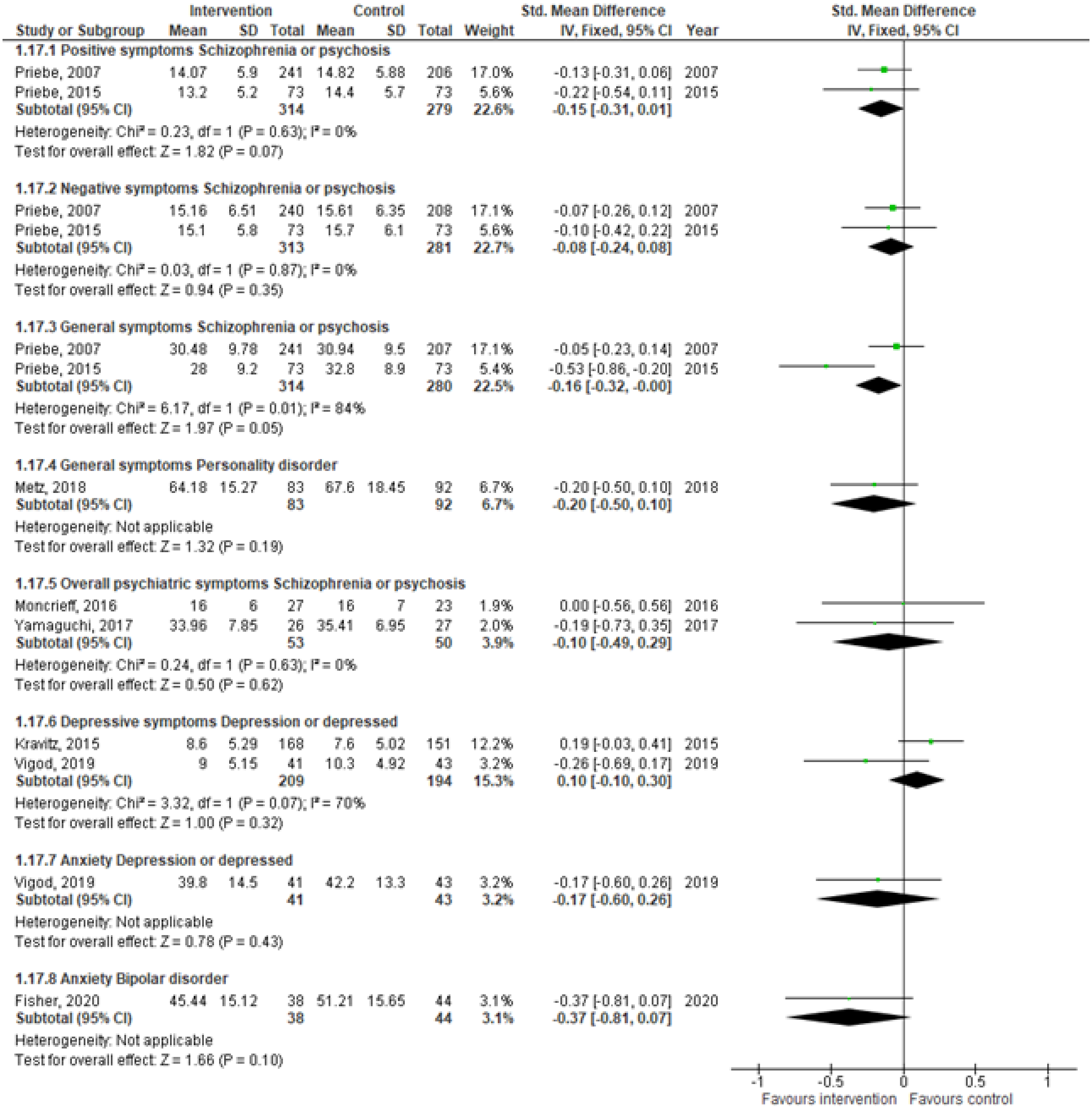

S.8. Forest plot on symptoms by diagnosis.
