# Supplementary material for: Digital Shared Decision-Making Interventions in Mental Healthcare: A Systematic Review and Meta-Analysis
Source: Front Psychiatry. 2021 Sep 6;12:691251. doi: 10.3389/fpsyt.2021.691251 (PMC8450495; doi:10.3389/fpsyt.2021.691251)
Supplement: Supplementary file 3 [file Data_Sheet_1.docx]

## Search string for EMBASE (adjusted to work for other databases)

(1 OR (2 AND 3) OR (2 AND 4) OR (3 AND 4)) AND 5 AND 6 AND 7

((shared decision* OR informed decision OR informed choice* OR share* information OR discuss* information OR discuss* decision*).ab,ti OR (((share* OR sharing* OR informed*).ti) AND ((decision* OR deciding* OR choice*).ti)))

(decision making OR decision process OR decision support technique* OR decision aid* OR decision support systems).ab,ti OR (choice behav* OR decision making* OR decision support* OR choice behave*).ab OR (decision*.ti AND (making* OR support* OR behav*).ti)

((patient participation* OR consumer participation* OR patient involvement* or consumer involvement*).ab OR ((patient* OR consumer* OR client*).ti AND (cooperat*.ab OR consensus.ab OR communication.ti OR collaborat*.ab OR concordance.ab OR negotiat*.ab OR discuss*.ab OR autonomy.ab OR preference.ab OR value.ab OR centered.ab OR involvement*.ab OR involving*.ab OR participation*.ab OR participating*.ab)))

((professional patient relations or working alliance or therapeutic alliance or ((nurses or physicians or nurse* or physician* or clinician* or doctor* or general practitioner* or health care professional* or healthcare professional* or health professional* or health provider* or health care provider* or healthcare provider* OR resident*).ti and (patients or patient* or consumer* or people* or person).ti)))

(Eating disorder OR Anorexia nervosa OR Bulimia OR Suici* OR Self mutilation OR Self injurious behav* OR Mood disorder OR Bipolar disorder OR Neurotic disorder OR Depressive disorder OR Dysthymic disorder OR depression OR affective disorder OR anxiety OR panic OR phobic disorder OR combat disorder OR stress disorder OR post traumatic OR Somatoform disorder OR Hypochondriasis OR Hysteria OR Conversion disorder OR munchausen syndrome OR Neurasthenia OR Fatigue syndrome OR Obsessive compulsive disorder OR Obsessive behav* OR Compulsive behavior OR paranoid disorder OR schizophrenia OR hebephreni* OR oligophreni* OR psychos* OR psychotic OR severe mental OR chronic mental OR mental illness OR mental disorder OR mental condition OR serious mental OR psychiat*)

(web portal OR webportal OR website or web site OR web based OR digital OR electronic OR smartphone OR smart phone OR mobile OR mobile health OR phone OR tablet OR eHealth OR e Health OR mHealth OR m Health OR telemedicine OR information tech* OR text messag* OR sms OR computer OR PC OR desktop OR desk top OR internet OR interactive OR computer assisted therapy OR virtual reality).ti,ab

(clinical trial or randomized controlled trial or randomized controlled trial or controlled clinical trial or double blind* or clinical trial* or pretest* or pre test* or posttest* or post test* or prepost*or pre post* or intervention*).ti,ab
